# RMAD1, a Novel Cell-Penetrating Peptide Derived from ADARB2: Preclinical Insights into Antigen Uptake and T Cell Activation

**DOI:** 10.7150/ijbs.132948

**Published:** 2026-06-04

**Authors:** Chaemin Lim, Chanho Park, Ee Chan Song, Yungyeong Shin, Wan Ki Kim, Yu Jin Park, Hyejoo Yoon, Cheolmin Ham, Sang Bum Kim, Seongmin Cho

**Affiliations:** 1 College of Pharmacy, CHA University, 335 Pangyo-ro, Bundang-gu, Seongnam-si, 13488 Gyeonggi-do, Republic of Korea.; 2 Remedi Co., Ltd. Research center, Songdo 21990, Korea.; 3 Institute for Rare Isotope Science, Institute for Basic Science, Daejeon 34000, Republic of Korea.; 4 College of Pharmacy, Sahmyook University, Seoul 01795, Korea.

**Keywords:** Cell-penetrating peptides, Peptide cancer vaccines, Lymph node retention, Antigen-specific T cell responses, IDDT platform

## Abstract

The development of effective cancer vaccines remains a major challenge in oncology, largely due to limited antigen delivery and suboptimal T cell priming. Here, we report RMAD1, a novel human-derived cell-penetrating peptide (CPP) originating from the ADARB2 (Adenosine Deaminase RNA Specific B2) protein, identified through an intra-dermal delivery technology (IDDT) platform, and evaluate its potential as a vaccine delivery enhancer. RMAD1 exhibited superior intracellular delivery compared with conventional CPPs and preferential uptake by antigen-presenting cells (APCs), including dendritic cells and macrophages. RMAD1 conjugated vaccines showed enhanced accumulation in draining lymph nodes and facilitated efficient antigen cross-presentation through the MHC class I pathway. In murine E.G7-OVA and TC-1 tumor models, RMAD1 conjugated vaccines induced robust antigen-specific CD8⁺ T cell responses across peripheral blood, lymphoid organs, and tumor tissues. Functional analyses revealed increased IFN-γ and TNF-α production by both CD8⁺ and CD4⁺ T cells, accompanied by a reduction in Foxp3⁺CD25⁺ regulatory T cells. In addition, RMAD1 conjugation promoted epitope spreading and established durable immunological memory, resulting in protection against tumor rechallenge. Therapeutic efficacy was further demonstrated in a TC-1 lung metastasis model, where RMAD1-based vaccination significantly reduced metastatic burden. Importantly, RMAD1-vaccinated mice exhibited therapeutic efficacy comparable to cisplatin treatment, while demonstrating a favorable safety profile. Together, these findings position RMAD1 as a next-generation CPP platform that outperforms existing peptides in enhancing antigen delivery and anti-tumor immunity, offering a promising strategy for advancing cancer vaccine development.

## Introduction

Cancer vaccines represent a powerful immunotherapeutic approach, aiming to generate antigen-specific immune responses that precisely target tumor-associated antigens 1-4. Despite substantial progress in identifying relevant tumor antigens and designing vaccine platforms, their clinical success has remained limited. One of the major bottlenecks lies in the inefficient activation of cytotoxic T lymphocytes (CTLs), primarily caused by poor antigen presentation and insufficient delivery to immune-inductive sites 5, 6.

Effective CD8⁺ T cell priming requires efficient antigen uptake, processing, and presentation by professional antigen-presenting cells (APCs), such as dendritic cells and macrophages, within the draining lymph nodes 7. However, conventional vaccine delivery exhibits poor lymph node delivery and limited intracellular accessibility, resulting in inadequate immune activation and reduced therapeutic benefit 8.

Cell-penetrating peptides (CPPs) have emerged as promising delivery tools capable of enhancing intracellular transport of various biological cargoes, including proteins, peptides, and nucleic acids 9-11. By facilitating endocytic pathways, CPPs can promote antigen processing through the MHC class I presentation pathway, a prerequisite for robust antigen-specific CD8⁺ T cells 12. Although CPP-conjugated vaccines exhibit improved lymph node accumulation and immune activation, commonly used CPPs such as TAT and R9 still suffer from limited delivery efficiency and inconsistent *in vivo* performance, underscoring the need for next-generation CPPs with enhanced bioactivity and safety.

In parallel, synthetic long peptides (SLPs) have gained attention as vaccine antigens as they require intracellular processing and contain both CD4⁺ and CD8⁺ T cell epitopes, leading to balanced and durable immune responses 13-17. However, the immunogenic potential of SLP based vaccines is highly dependent on effective delivery to APCs, underscoring the importance of optimized delivery systems 18-20.

Here, we report RMAD1, a novel human-derived CPP identified through our intra-dermal delivery technology (IDDT) platform, as an efficient delivery vehicle for cancer vaccines. RMAD1 demonstrates superior intracellular delivery compared with conventional CPPs and promotes preferential antigen uptake by APCs within draining lymph nodes. Using E.G7-OVA and TC-1 tumor models, we show that RMAD1-based vaccines enhance antigen cross-presentation, induce robust antigen-specific T cell responses, and confer durable anti-tumor immunity. These findings establish RMAD1 as a promising next-generation CPP platform for improving cancer vaccine efficacy.

## Results

### Identification of a novel cell-penetrating peptide using the IDDT platform

**Figure 1:**
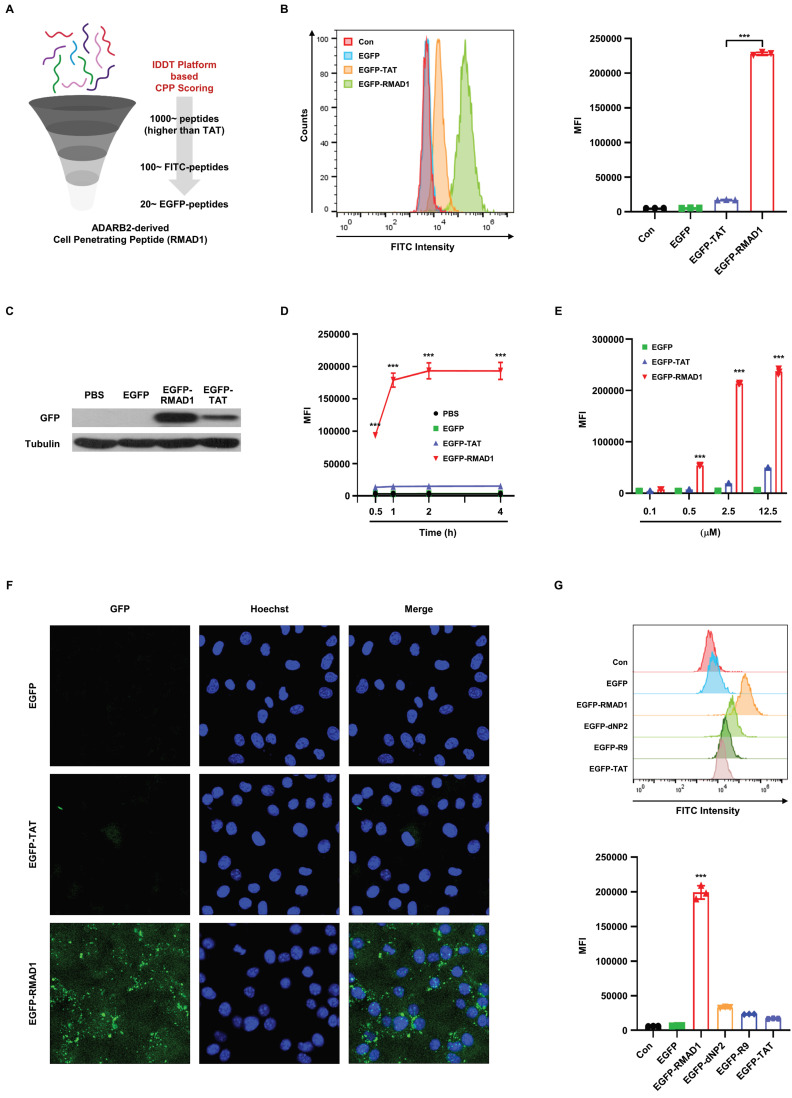
** Identification of a novel cell-penetrating peptide using the IDDT platform.** (A) Schematic overview of the screening process for cell-penetrating peptides screening using IDDT platform. (B) HaCaT cells were incubated with 2.5 μM FITC-conjugated peptides for 2 h. The mean fluorescence intensity (MFI) was measured by flow cytometry, and FITC-conjugated TAT was used as a reference for penetration efficiency (n=3 per group). (C) HaCaT cells were treated with 1 μM EGFP, EGFP-RMAD1, or EGFP-TAT for 2 h and analyzed by western blotting. Penetration ability was assessed using anti-GFP antibodies, with tubulin serving as the loading control (n=3 per group). (D, E) HaCaT cells were treated with the indicated recombinant proteins in a time (D) and dose (E) dependent manner. MFI was quantified by flow cytometry (n=3 per group). (F) Confocal microscopy images of HaCaT cells treated with indicated fusion proteins. Cells were incubated with 2.5 μM of recombinant proteins for 2 h before imaging. Hoechst 33342 was used for nuclei staining. (G) HaCaT cells were treated with 2.5 μM EGFP, EGFP-RMAD1, or other EGFP-CPP fusion proteins for 2 h, and MFI was measured by flow cytometry (n=3 per group) and representative histograms are shown. Data are presented as mean ± SD. Statistical significance was determined by student's t-test (*** p < 0.001).

To identify novel cell-penetrating peptides (CPPs) for targeted delivery, we employed our "intra-dermal delivery technology" (IDDT). This platform was designed to facilitate the efficient penetration of therapeutic agents 21, 22. IDDT is a rational peptide prioritization system designed to rank peptides based on physicochemical and sequence-derived properties known to govern CPP performance, including net cationic charge, amphipathic balance and structural flexibility. Candidate peptides were assigned CPP scores calculated as composite indices integrating normalized physicochemical parameters, allowing relative ranking of candidate peptides. Peptides exceeding the score of a reference CPP (TAT) were prioritized for further analysis. In addition, sequence-based scoring metrics, including SVM-derived scores, were used as supportive parameters to assist candidate prioritization. The scoring framework was further refined by incorporating experimentally measured cellular uptake data, enabling feedback-driven improvement of peptide prioritization. Through this approach, we identified over 1,000 peptides with potential cell-penetrating activity. From these, the top 100 peptides with higher scores than TAT were selected for experimental validation. Each peptide was initially conjugated with FITC to evaluate cellular uptake, and the top 20 candidates were further engineered as enhanced green fluorescent protein (EGFP) fusion constructs to enable quantitative comparison of intracellular delivery efficiency. Among these candidates, RMAD1, a peptide derived from the human protein ADARB2, exhibited the highest intracellular fluorescence intensity compared to other CPPs (Figure 1A).

This initial screening result was independently validated using flow cytometry, which demonstrated that EGFP-RMAD1 achieved significantly higher intracellular fluorescence intensity compared with EGFP alone or EGFP-TAT (Figure 1B). Consistently, western blot analysis demonstrated enhanced intracellular accumulation of EGFP-RMAD1 compared with EGFP-TAT (Figure 1C). Notably, the delivery efficiency of EGFP-RMAD1 exhibited both time- and dose-dependent characteristics, reinforcing its potential as a robust delivery vehicle (Figure 1D-E and Figure S1A-B). To further quantify the penetration efficiency, a dose response uptake assay was performed, and EC₅₀ (0.8719 μM) was calculated based on mean fluorescence intensity (Figure S1C). Moreover, immunofluorescence assay confirmed robust intracellular localization of EGFP-RMAD1 compared with other CPPs (Figure 1F). Finally, RMAD1 was compared with additional well characterized CPPs, including R9, and dNP2. By connecting other CPPs into our EGFP expression system, we found that our RMAD1 has shown the greatest internalization efficiency among other CPPs, indicating RMAD1 can work as highly effective CPP for macromolecule delivery (Figure 1G).

To further validate that the observed fluorescence originated from intracellular localization rather than membrane-associated binding, we performed trypan blue quenching in selected experiments. Under these conditions, RMAD1 retained strong fluorescence signals, confirming that the majority of the signal resulted from true internalization (Figure S1D). This efficient intracellular delivery may be associated with intrinsic structural features of RMAD1, as circular dichroism (CD) analysis revealed a predominantly α-helical conformation under membrane-mimicking conditions (Figure S2A-B). To further address whether the observed delivery is specific to RMAD1 or generalizable to other peptide sequences, we evaluated a panel of RMAD1-derived and sequence-modified variants. Peptides with altered sequence composition, including arginine-to-lysine substitutions and terminal truncations, exhibited significantly reduced uptake compared to the original RMAD1, indicating that efficient intracellular delivery is not a general property of cationic peptides but depends on specific sequence features (Figure S1E).

In addition, to extend these findings beyond EGFP systems, we assessed the intracellular delivery of SOD1 as an endogenous protein cargo. Flow cytometry and western blot analyses confirmed enhanced intracellular accumulation of SOD1 in the RMAD1-conjugated group (Figure S3A-B). Importantly, in an LPS-induced DC2.4 model, SOD1-RMAD1 significantly reduced TNF-α levels, indicating that the delivered protein retained functional activity after internalization (Figure S3C).

### RMAD1 enhances lymph node delivery and APC uptake via energy-dependent endocytosis

**Figure 2:**
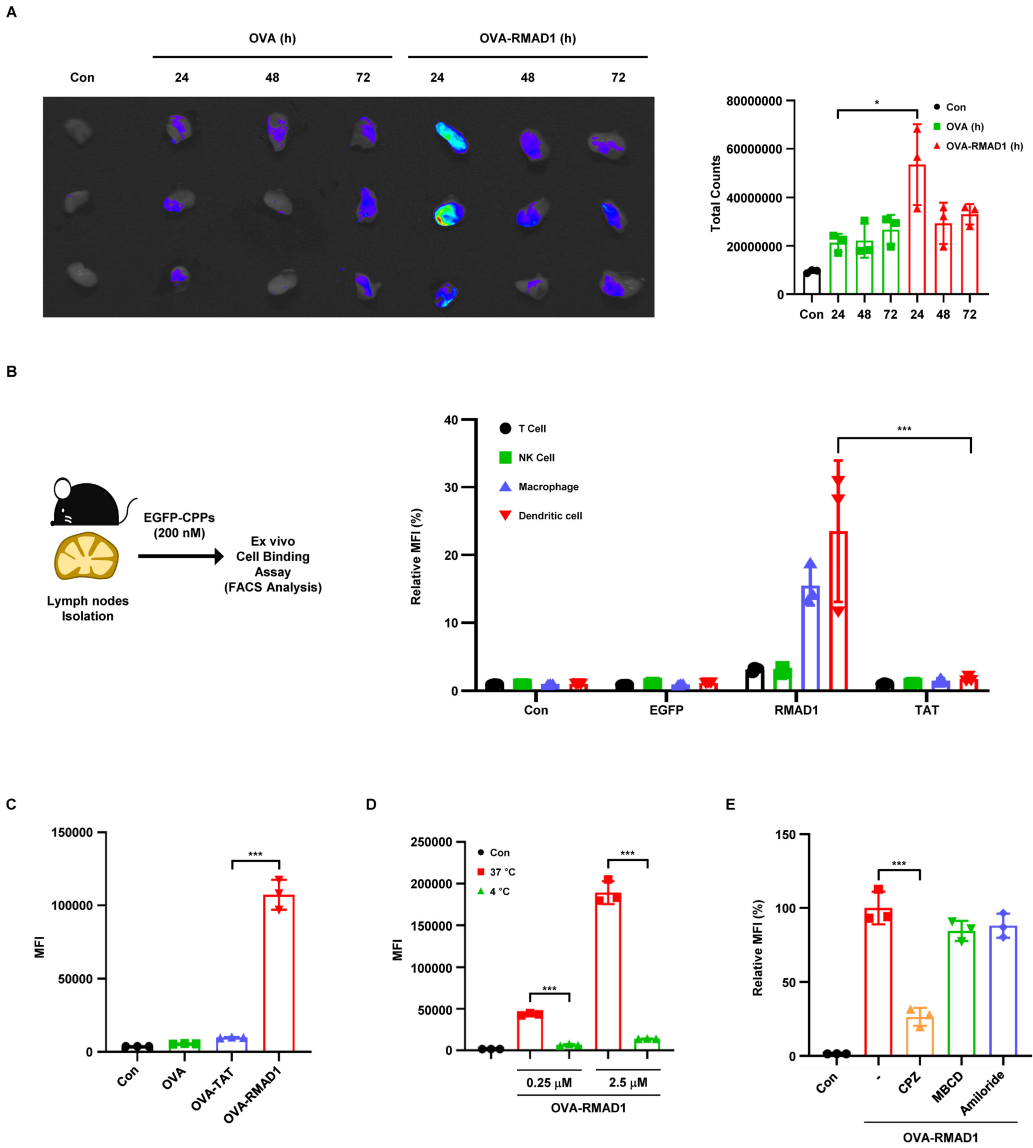
** RMAD1 enhances lymph node delivery and APC uptake via energy-dependent endocytosis.** (A) C57BL/6 mice were subcutaneously injected with 25 nmol of FITC-conjugated OVA-RMAD1. Draining lymph nodes were harvested at 24, 48, and 72 h post-administration, and peptide accumulation was visualized by IVIS imaging. Fluorescence signals were quantified as total fluorescence counts to assess time-dependent lymph node retention (n=3 per group). (B) Lymph nodes from naïve C57BL/6 mice were excised and incubated *ex vivo* with EGFP, EGFP-TAT, or EGFP-RMAD1 for 2 h. Cellular uptake efficacy was assessed by flow cytometry by gating on T, NK, macrophage, and dendritic cells, and MFI was measured (n=3 per group). (C) DC2.4 cells were treated with 2.5 μM of FITC-conjugated peptides for 2 h. MFI was analyzed by flow cytometry, using FITC-TAT as a reference control for penetration efficiency (n=3 per group). (D) Quantitative analysis of RMAD1 uptake in DC2.4 cells at different concentrations under 4 °C and 37 °C conditions (n=3 per group). (E) DC2.4 cells were pre-treated with specific endocytosis inhibitors, followed by treatment with OVA-RMAD1 for 2 h. Relative MFI was determined by flow cytometry (n=3 per group). Data are presented as mean ± SD and statistical significance was assessed by student's t-test (* p < 0.05, *** p < 0.001).

Recently, various studies have been shown that CPPs can be used as a delivery tool for increasing vaccine efficacy by enhancing lymph node retention and antigen uptake 12, 23. Notably, CPPs such as TAT and R9, which have been shown to enhance lymph node targeting, also score highly within the IDDT framework. This observation supports the hypothesis that IDDT selected peptides are enriched for features favorable for *in vivo* antigen delivery. To evaluate whether RMAD1 improves lymph node retention, RMAD1 was conjugated to the ovalbumin synthetic long peptide OVA247-264, which efficiently activates both CD4⁺ and CD8⁺ T cells and induces antitumor immune responses 24, 25. Fluorescence signals in draining lymph nodes were evaluated at 24, 48, and 72 h following administration. The OVA-RMAD1 group exhibited the highest fluorescence intensity at 24 h, followed by a gradual decrease at later time points (Figure 2A), indicating efficient lymphatic delivery. Consistent with this kinetic profile, supplementary biodistribution analysis revealed detectable fluorescence signals in the kidney at 24 h (Figure S4A), suggesting that OVA-RMAD1 is likely cleared through systemic circulation rather than prolonged nonspecific tissue accumulation.

To further explore the mechanism of CPP uptake, we performed *ex vivo* uptake assays on isolated inguinal lymph nodes from C57BL/6 mice. We used EGFP-tagged CPPs for *ex vivo* uptake assays and we found that RMAD1-conjugated EGFP was preferentially taken up by antigen-presenting cells, particularly macrophages and dendritic cells, whereas unconjugated or TAT-conjugated showed limited uptake (Figure 2B and Figure S5A). Consistent with these findings, RMAD1 significantly enhanced antigen uptake in DC2.4 dendritic cells compared with OVA alone or TAT-conjugated OVA (Figure 2C and Figure S5B).

To determine whether RMAD1 uptake is mediated by an energy-dependent endocytic process, uptake assays were performed at 37 °C and 4 °C in DC2.4 cells. Cellular uptake of RMAD1 was markedly reduced at 4 °C, indicating that RMAD1 internalization is predominantly mediated by energy-dependent endocytosis (Figure 2D and Figure S5C). To further dissect the cellular uptake pathway, we treated the cells with inhibitors such as CPZ, MBCD, and Amiloride 26. The diminished uptake of OVA-RMAD1 following CPZ treatment indicated that RMAD1 likely penetrates cells via clathrin-mediated endocytosis (Figure 2E and Figure S5D). Consistent with these findings, temperature-dependent uptake profiles were observed in non-APC cell types, including TC-1 tumor cells and HaCaT keratinocytes (Figure S5E-F), indicating that RMAD1 utilizes a general endocytic mechanism. Importantly, RMAD1 treatment did not induce detectable cytotoxicity in TC-1, DC2.4 or HaCaT cells, as confirmed by long-term viability assays and Annexin V/PI staining, indicating that enhanced cellular uptake was not associated with membrane disruption or cell death (Figure S6A-B).

### RMAD1 conjugation enhances cross-presentation to promote T cell priming and anti-tumor immune responses

**Figure 3:**
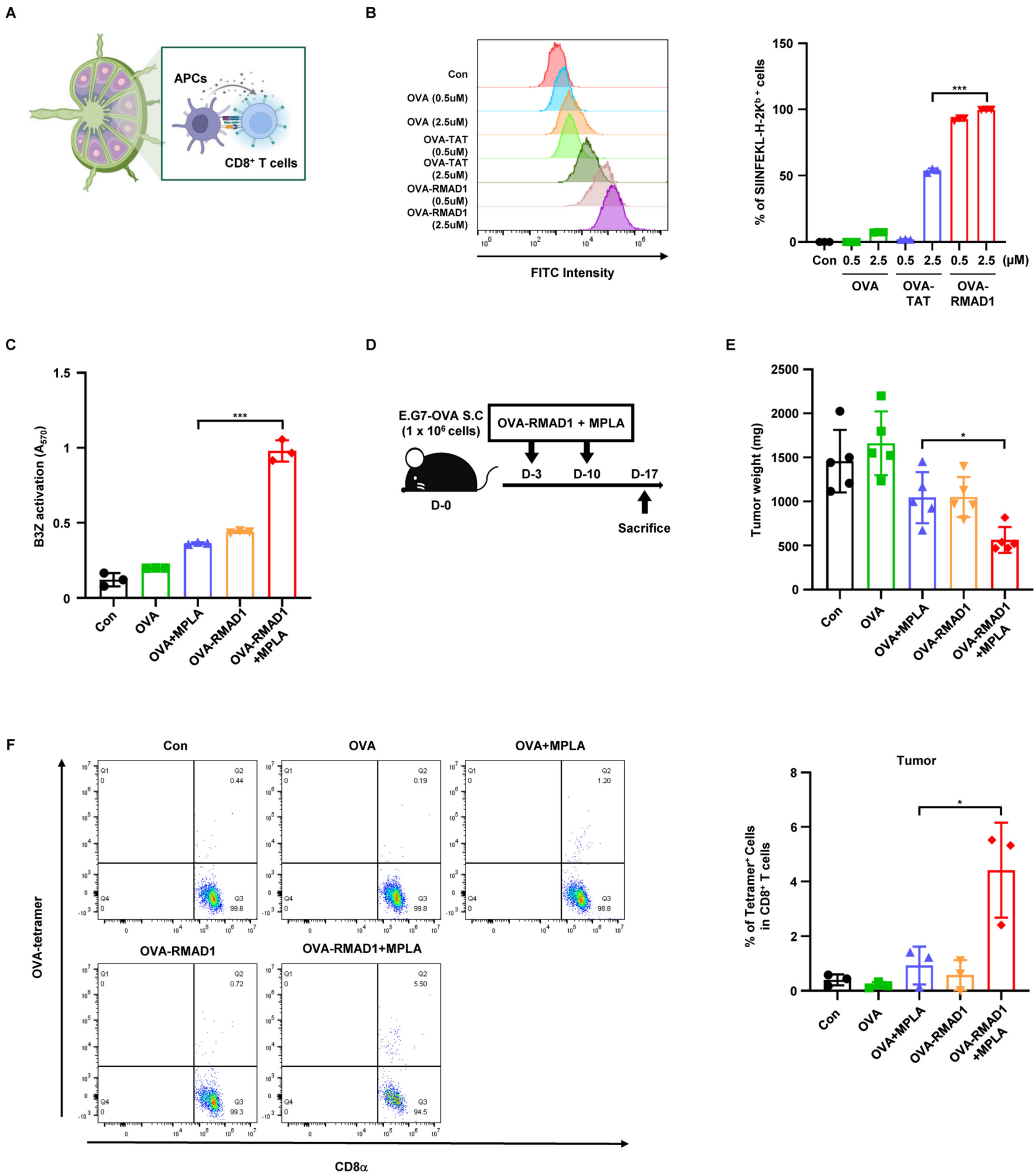
** RMAD1 conjugation enhances cross-presentation to promote T cell priming and anti-tumor immune responses.** (A) Schematic illustration of enhanced vaccine efficacy using the novel CPP, RMAD1. (B) DC2.4 cells were treated with OVA, OVA-RMAD1, or OVA-TAT at indicated concentrations for 24 h. Percentage of surface presentation of SIINFEKL-H-2Kb positive cells were quantified by flow cytometry (n=3 per group). (C) DC2.4 cells were pulsed with the indicated antigens in the presence of MPLA adjuvant and co-cultured with B3Z cells. T cell activation was measured after 24 h by CPRG assay, with absorbance read at 570 nm (n=3 per group). (D, E) Mice bearing E.G7-OVA tumors were vaccinated subcutaneously on days 3 and 10 post-tumor implantation. Schematic overview of the experimental schedule is shown (D) and tumor weights (E) were measured on day 17 after sacrifice (n=5 per group). (F) Tumor-infiltrating lymphocytes were analyzed by flow cytometry, quantifying OVA-specific CD8+ T cells and representative dot plots are shown (n=3 per group). The results are presented as mean ± SD. Statistical significance was analyzed by student's t-test (* p < 0.05, *** p < 0.001).

To determine whether RMAD1-mediated antigen delivery enhances antigen processing and presentation, DC2.4 dendritic cells were treated with OVA, OVA-TAT, or OVA-RMAD1, and the level of MHC class I-bound SIINFEKL-H-2Kb complexes was measured by flow cytometry. Compared with both the antigen alone and the TAT-conjugated counterpart, OVA-RMAD1 treatment resulted in a significantly higher proportion of MHC class I SIINFEKL positive cells, indicating enhanced antigen cross-presentation at the population level (Figure 3A-B).

To assess whether this enhanced presentation translated into functional T cell priming, DC2.4 cells pulsed with each antigen were co-cultured with B3Z T cell hybridoma cells in the presence of the MPLA adjuvant. T cell activation was quantified using the CPRG assay, which measures β-galactosidase activity as a surrogate for TCR signaling (Figure 3C) 27. OVA-RMAD1-pulsed DCs induced significantly higher T cell activation compared to other groups, demonstrating that RMAD1 improves the functional priming capacity of dendritic cells 28.

We next assessed the anti-tumor efficacy of RMAD1-conjugated antigen *in vivo* using the E.G7-OVA tumor model. Mice were vaccinated with OVA alone, OVA + MPLA, OVA-RMAD1, or OVA-RMAD1 + MPLA, administered on days 3 and 10 after tumor implantation (Figure 3D) 29, 30. Among these groups, vaccination with OVA-RMAD1 + MPLA resulted in the most significant reduction in tumor burden (Figure 3E). Consistently, flow cytometry analysis revealed a significantly increased frequency of OVA-specific CD8⁺ T cells infiltrating the tumor microenvironment in the RMAD1-conjugated groups, particularly when combined with MPLA (Figure 3F). These results demonstrate that RMAD1 enhances the therapeutic efficacy of peptide vaccination in the presence of adjuvant stimulation, leading to robust tumor-specific T cell responses *in vivo*.

### Conjugation of E7 SLP to RMAD1 enhances vaccine potency

**Figure 4:**
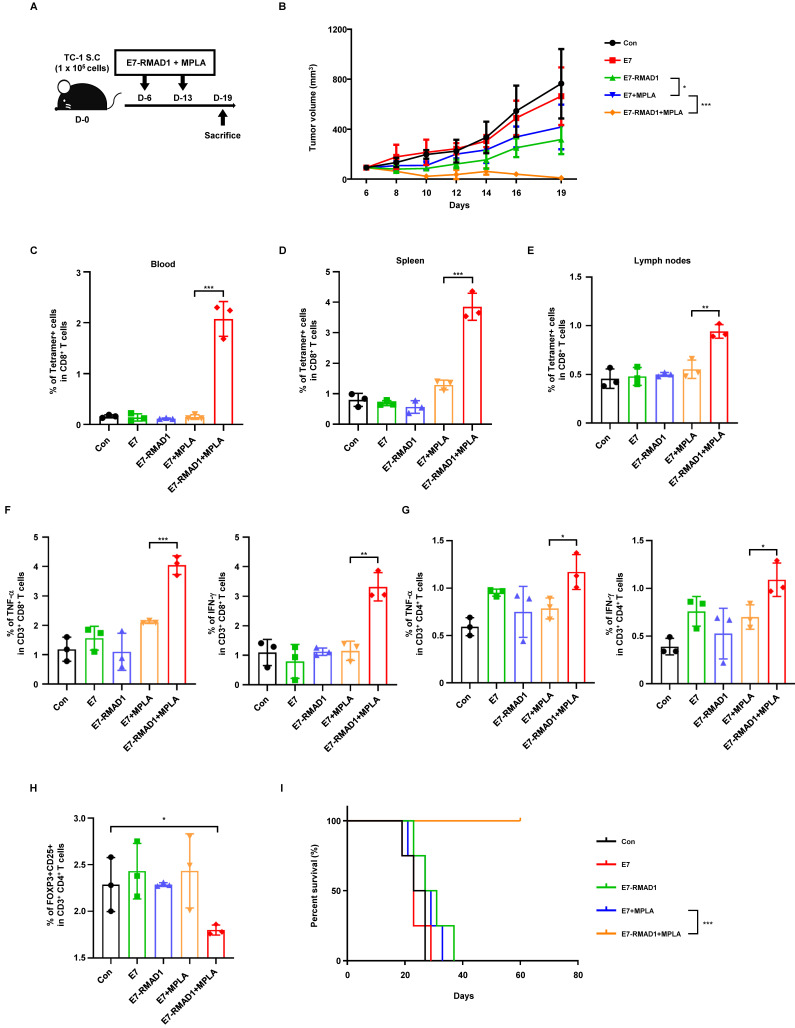
** Conjugation of E7 SLP to RMAD1 enhances vaccine potency.** (A, B) C57BL/6 mice were subcutaneously implanted with TC-1 cells and vaccinated with E7 SLP-RMAD1 on days 6 and 13 post-implantation. Schematic representation of the vaccination schedule is shown (A) and tumor volumes (B) were measured at indicated time points (n=5 per group). (C) On day 19, PBMCs were isolated from tumor-bearing mice, and the frequency of E7-specific CD8+ T cells were assessed by flow cytometry (n=3 per group). (D, E) E7-specific CD8⁺ T cell responses were further evaluated in the spleen (D) and draining lymph nodes (E) by tetramer staining (n=3 per group). (F, G) Intracellular cytokine staining was performed to assess TNF-α and IFN-γ production by CD8⁺ (F) and CD4⁺ T cells (G) in the spleen (n=3 per group). (H) The frequency of Foxp3⁺CD25⁺ regulatory T cells in peripheral blood was analyzed by flow cytometry (n=3 per group). (I) Mice with tumor volumes exceeding 1500 mm3 were euthanized, and survival curves were generated (n=4 per group). The results are presented as mean ± SD and the statistical significance was analyzed by student's t-test (* p < 0.05, ** p < 0.01, *** p < 0.001). For the survival curve, statistical analysis was performed using Kaplan-Meier with the log-rank (Mantel-Cox) test.

To evaluate the therapeutic potential of RMAD1 in a clinically relevant setting, RMAD1 was conjugated to an E7 derived synthetic long peptide (SLP) and tested in the TC-1 tumor model. C57BL/6 mice bearing subcutaneous TC-1 tumors were vaccinated with E7-RMAD1 on days 6 and 13 post-implantation. Vaccination with E7-RMAD1 significantly delayed tumor progression, with the most pronounced tumor growth inhibition observed when combined with the MPLA adjuvant (Figure 4A-B).

To further assess the systemic immune response elicited by the vaccine, peripheral blood mononuclear cells (PBMCs) were collected on day 19 post-vaccination and analyzed by flow cytometry. E7-specific CD8⁺ T cells were quantified using a PE-conjugated MHC class I tetramer loaded with the E7 epitope peptide. Co-administration of E7-RMAD1 with MPLA induced a marked expansion of E7-specific CD8⁺ T cells in peripheral blood, spleen, and draining lymph nodes compared with control or single-component treatment groups (Figure 4C-E and Figure S7A-C), indicating robust T cell priming across different lymphoid organs.

To further characterize the functional quality of vaccine-induced T cell responses, intracellular cytokine staining was performed. In the spleen, both CD8⁺ and CD4⁺ T cells from the E7-RMAD1 + MPLA group exhibited significantly increased production of TNF-α and IFN-γ compared with control groups (Figure 4F-G and Figure S8A-B), indicating the induction of a strong type 1 effector T cell response. Within the tumor microenvironment, E7-RMAD1 + MPLA treatment resulted in the highest proportion of IFN-γ⁺ CD8⁺ tumor-infiltrating lymphocytes (Figure S9A).

Immunoregulatory effects were further evaluated in peripheral blood. E7-RMAD1 + MPLA vaccination significantly reduced the frequency of Foxp3⁺CD25⁺ regulatory T cells (Figure 4H and Figure S10A), while no significant changes were observed in CD8⁺ T cell exhaustion markers, including PD-1⁺LAG-3⁺ or PD-1⁺TIM-3⁺ populations (Figure S11A-B), indicating enhanced immune activation without induction of T cell exhaustion.

Consistent with these immunological findings, Kaplan-Meier survival analysis demonstrated that the E7-RMAD1 + MPLA group achieved the greatest survival benefit, with a substantial proportion of mice remaining tumor-free beyond the experimental endpoint, whereas control mice were euthanized due to excessive tumor burden (Figure 4I). Collectively, these results demonstrate that conjugation of RMAD1 to E7 SLP markedly enhances vaccine potency by promoting robust functional antigen-specific T cell responses, reducing immunosuppressive Treg populations, and translating into durable tumor control and survival benefit.

### RMAD1 conjugated vaccination elicits durable anti-tumor immunity in primary and metastatic models

**Figure 5:**
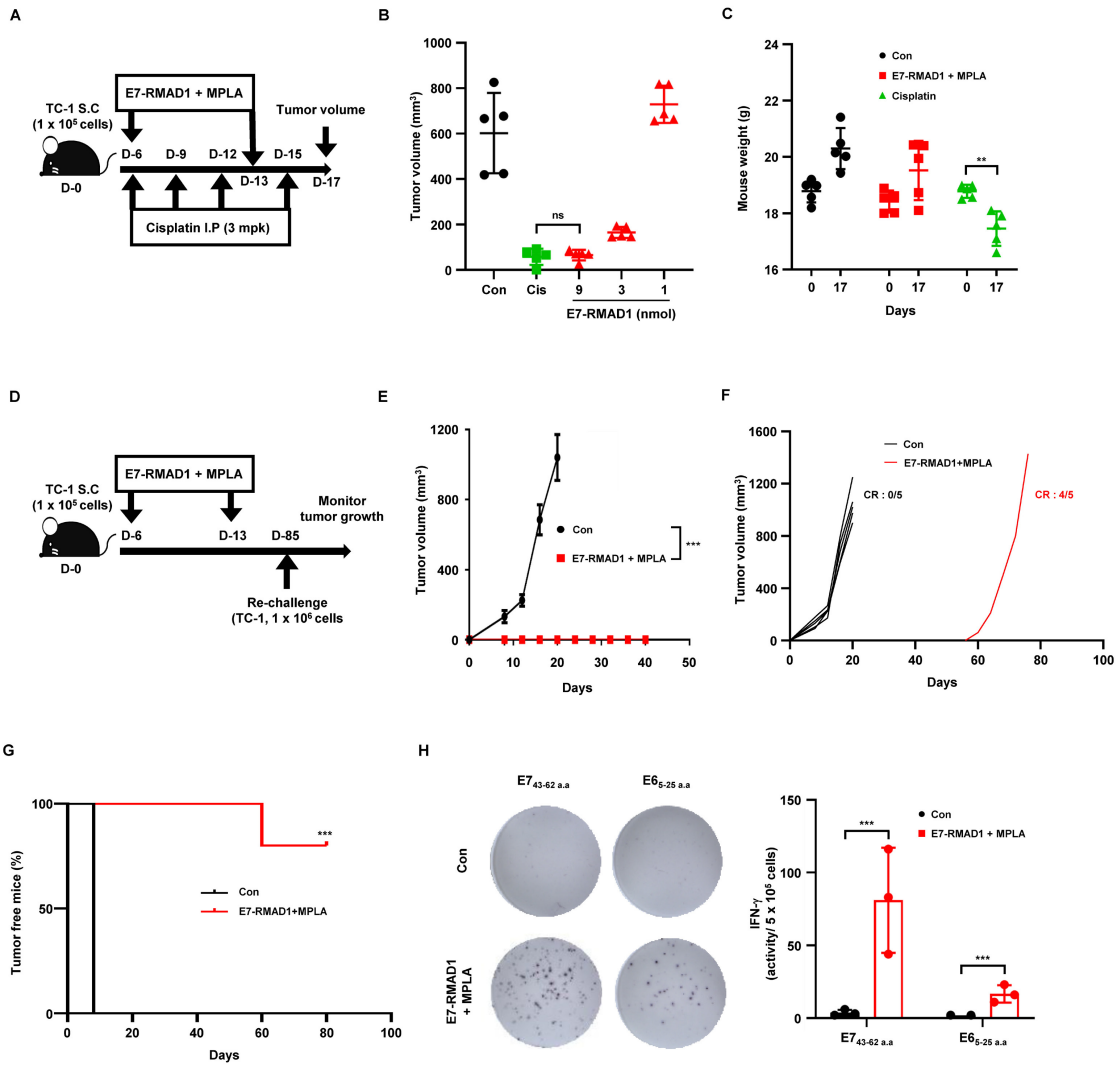
** RMAD1 conjugated vaccination elicits durable anti-tumor immunity with immune memory and evidence of antigen spreading.** (A-C) Therapeutic vaccination scheme and antitumor efficacy in a TC-1 tumor model. TC-1 tumor bearing mice were treated with E7-RMAD1 plus MPLA on days 6 and 13, or with cisplatin (3 mg/kg) on days 6, 9, 12, and 15 (A). Tumor volume (B) and body weight (C) were measured on day 17 (n = 5 per group). (D-G) Tumor-free mice were re-challenged with 1 × 10⁶ TC-1 cells on day 85, and subsequent tumor growth was monitored (n = 5 per group), including schematic illustration of the experimental design (D), tumor volume following re-challenge (E), individual tumor growth curves with complete response (CR) indicated (F), and tumor-free status was analyzed using Kaplan-Meier curves (G), demonstrating prolonged tumor-free status and delayed tumor onset in the vaccinated group. (H) Antigen spreading was assessed by re-stimulating immune cells with E6 or E7 peptides followed by IFN-γ ELISPOT analysis (n=3 per group). Data are presented as mean ± SD. Statistical significance was determined using Student's t-test (**p < 0.01, ***p < 0.001). Survival curves were analyzed using the Kaplan-Meier method with the log-rank (Mantel-Cox) test.

To further investigate the therapeutic efficacy and long-term immunological impact of RMAD1-conjugated vaccines, we conducted a series of *in vivo* experiments using the TC-1 tumor model. Given that cisplatin is widely employed as a first-line chemotherapeutic agent for cervical cancer, we first compared the anti-tumor activity and systemic toxicity of the E7-RMAD1 peptide vaccine with that of cisplatin (Figure 5A) 31, 32. Mice with established TC-1 tumors were treated with E7-RMAD1 plus the MPLA adjuvant on days 6 and 13 post-implantation. A control group received cisplatin at 3 mg/kg on days 6, 9, 12, and 15. By day 17, tumor volume analysis revealed that the E7-RMAD1 + MPLA group achieved comparable tumor suppression to that of the cisplatin group (Figure 5B). However, cisplatin-treated mice experienced significant weight loss, indicating systemic toxicity, whereas E7-RMAD1 vaccinated mice maintained stable body weight throughout the treatment period (Figure 5C). This comparison suggests that RMAD1-based immunotherapy provides effective tumor control with reduced adverse effects. Importantly, cisplatin was included as a clinically relevant benchmark control, and this comparison was intended to provide contextual reference rather than to imply direct therapeutic equivalence between chemotherapy and vaccine-based immunotherapy.

To determine whether RMAD1-conjugated vaccination generated long-term immune memory capable of protecting against future tumor encounters, we performed a tumor re-challenge experiment. On day 85 post-initial vaccination, mice that had completely cleared tumors were re-inoculated subcutaneously with 1 × 10⁶ TC-1 cells. In parallel, age-matched naïve mice received the same tumor cell inoculation as a control. Strikingly, E7-RMAD1-vaccinated mice exhibited strong protection against tumor re-challenge, showing markedly suppressed tumor growth compared to naïve controls (Figure 5D-E). While all naive mice rapidly developed tumors, vaccinated mice maintained effective tumor control during the early phase following re-challenge. Consistent with this, individual tumor growth curves demonstrated sustained tumor suppression in most vaccinated animals, with only a single mouse showing delayed tumor development (Figure 5F). To further evaluate long-term tumor progression, tumor-free status over time was analyzed using Kaplan-Meier curves, which revealed a significant delay in tumor onset and a substantial proportion of mice remaining tumor-free (Figure 5G). To further evaluate the breadth of the immune response induced by the vaccine, we examined antigen spreading a process wherein immune responses extend beyond the initial vaccine antigen to other tumor-associated epitopes. Tumor-free mice from the vaccinated group were euthanized, and immune cells were restimulated *ex vivo* with either the E7 peptide used for vaccination or a non-vaccine HPV-derived antigen, E6. IFN-γ ELISPOT analysis revealed strong T cell responses to both E7 and E6 peptides, indicating that RMAD1-mediated vaccination had triggered epitope spreading to additional tumor-associated antigens (Figure 5H).

**Figure 6:**
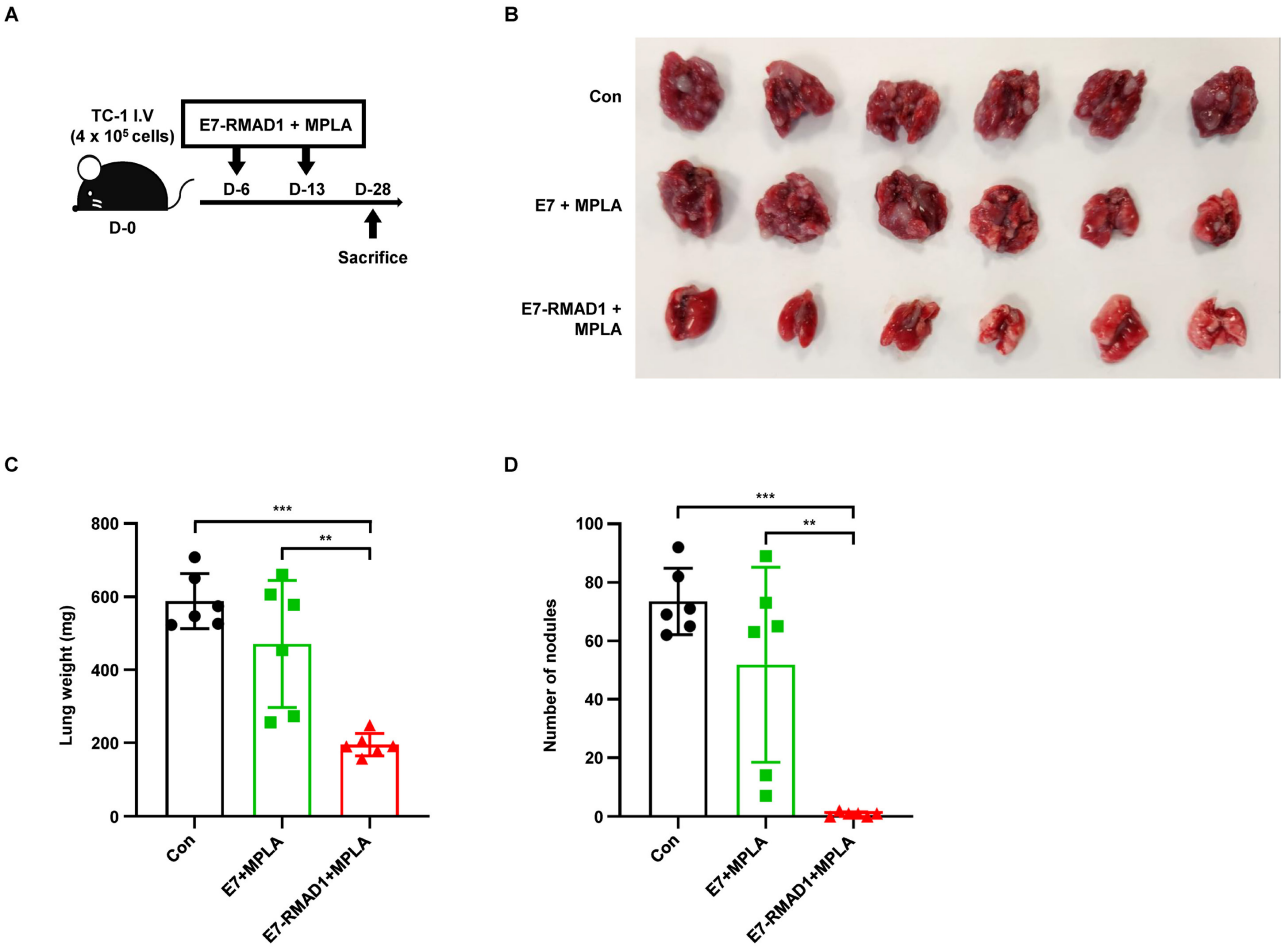
** RMAD1 conjugated vaccination suppresses lung metastasis in a TC-1 tumor model.** (A-D) Schematic illustration of the therapeutic vaccination protocol using a TC-1 lung metastasis mouse model (A). At day 28 after tumor challenge, lungs were harvested and images were acquired (B). Lung weight (C) and the number of metastatic nodules (D) were quantified (n=6 per group). Data are presented as mean ± SD. Statistical significance was determined using Student's t-test (** p < 0.01, *** p < 0.001).

We next examined the therapeutic potential of RMAD1-based vaccination in a metastatic setting. Lung metastasis was induced by intravenous injection of TC-1 cells, followed by vaccination with E7-RMAD1 on days 6 and 13 (Figure 6A). Mice were sacrificed on day 28, and vaccinated mice showed reduced metastatic lung burden, as indicated by lower lung weights and fewer metastatic nodules compared with controls (Figure 6B-D). Collectively, these results demonstrate that RMAD1-conjugated vaccines elicit potent anti-tumor immunity across both primary and metastatic tumor settings, induce durable immune memory, and promote epitope spreading.

### Systemic toxicity and safety evaluation of RMAD1-conjugated vaccine

To evaluate the systemic toxicity and inflammatory potential of RMAD1-conjugated vaccines, C57BL/6 mice were treated with control, cisplatin, Pam2CSK4, or E7-RMAD1. Cisplatin (15 mg/kg) and Pam2CSK4 (100.6 nmol) were included as positive controls for cytotoxicity and innate immune driven inflammation, respectively. E7-RMAD1 was administered at a dose of 51.8 nmol, corresponding to more than five-fold higher than the established efficacious dose, to rigorously assess the safety margin.

**Figure 7:**
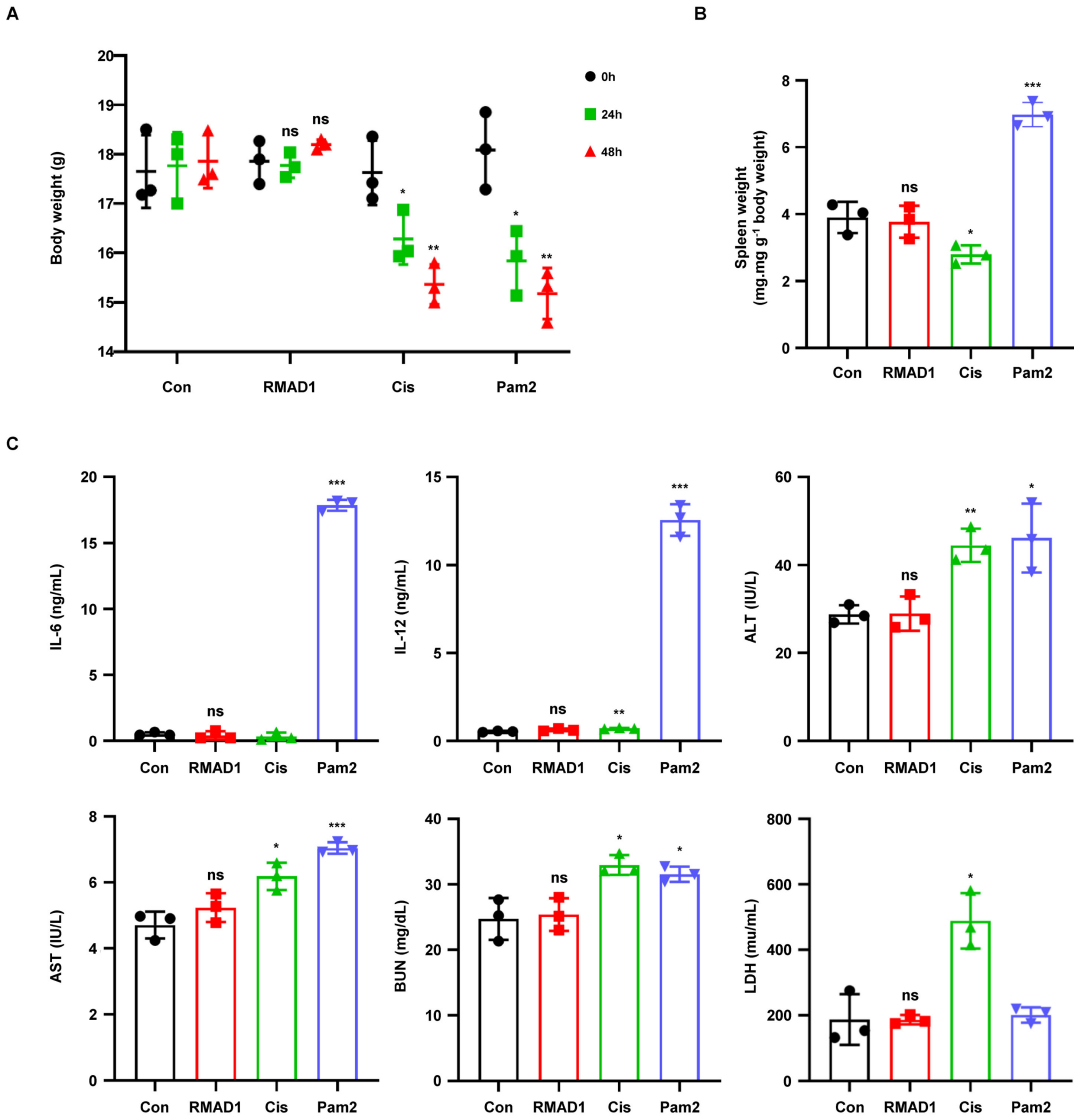
** Systemic toxicity and safety evaluation of RMAD1-conjugated vaccine.** (A) C57BL/6 mice were administered with control, cisplatin, Pam2CSK4, or RMAD1-conjugated vaccines. Schematic representation of treatment schedule and body weight changes were monitored (n=3 per group). (B) Spleen weights were measured at the endpoint and normalized to body weight (n=3 per group). (C) Serum cytokine levels (IL-6 and IL-12) and clinical chemistry parameters, including liver function markers (ALT and AST), kidney function marker (BUN), and tissue damage marker (LDH) were evaluated (n=3 per group). Data are presented as mean ± SD. Statistical significance was determined by Student's t-test (*p < 0.05, ** p < 0.01, ***p < 0.001).

Body weight was monitored over time to assess systemic toxicity. While mice treated with cisplatin or Pam2CSK4 exhibited measurable body weight loss, no significant changes in body weight were observed in the E7-RMAD1 treated group compared with control mice (Figure 7A). Consistent with this finding, the spleen weight to body weight ratio, a surrogate marker of systemic inflammation, remained unchanged in the E7-RMAD1 group, whereas a clear increase was observed in cisplatin and Pam2CSK4-treated mice (Figure 7B).

To further evaluate inflammatory responses and organ toxicity, serum cytokine levels and clinical chemistry parameters were analyzed. No significant elevations in pro-inflammatory cytokines, including IL-6 and IL-12, were detected in mice treated with E7-RMAD1 (Figure 7C). In addition, liver function markers (ALT and AST), the kidney function marker BUN, and the tissue damage marker LDH showed no significant differences between the E7-RMAD1 and control groups, whereas cisplatin or Pam2CSK4 treatment induced marked alterations in these parameters (Figure 7C).

Collectively, these results demonstrate that RMAD1-conjugated vaccines do not induce detectable systemic toxicity or inflammatory responses, even at doses substantially exceeding the efficacious range, supporting the favorable *in vivo* safety profile of E7-RMAD1.

## Discussion

In this study, we have identified and characterized RMAD1 as a novel and highly effective cell-penetrating peptide (CPP) with significant potential for targeted drug delivery and immunotherapy applications. Our findings demonstrate that RMAD1 exhibits superior cellular uptake and internalization efficiency compared to established CPPs, such as the TAT and R9 peptide, highlighting its promise as a delivery vehicle for macromolecules, including proteins, antigens, and vaccines 33, 34.

Further investigations into the antigen delivery capability of RMAD1 highlight its effectiveness in the context of immune system activation. By conjugating RMAD1 with synthetic OVA247-264EAAAAKSL SLP, we observed significantly improved antigen uptake in lymph nodes, crucial for initiating adaptive immune responses 35, 36. This suggests that RMAD1 has the ability to efficiently deliver antigens to lymphoid tissues, which is essential for the activation of T cells and the induction of robust immune responses. The preferential uptake of RMAD1-conjugated EGFP by antigen-presenting cells (APCs), particularly macrophages and dendritic cells, underscores the targeting specificity of RMAD1. Especially, dendritic cells are critical for initiating T cell responses, making RMAD1 a promising candidate for enhancing vaccine efficacy 37, 38.

Our data also highlight superior antigen delivery efficacy compared to the conventional CPPs, with fluorescence measurements showing increased penetration of antigen-presenting cells and improved immunogenicity. This is particularly important in vaccine development, where the efficiency of antigen delivery can greatly impact the overall effectiveness of the immunization strategy 39, 40. The observed clathrin-mediated endocytosis mechanism underlying cellular uptake of RMAD1 provides insight into the molecular processes driving its superior delivery capabilities. Understanding these internalization pathways will be essential for optimizing RMAD1-based delivery systems for various therapeutic contexts.

In cancer immunotherapy, conjugating RMAD1 with synthetic long peptides derived from the E7 oncoprotein (E743-62) demonstrated promising results in preclinical tumor models. In the TC-1 tumor model, E7-RMAD1 vaccination led to a significant reduction in tumor growth, accompanied by an increase in antigen-specific T cell responses. These responses were not restricted to a single compartment but were consistently observed across peripheral blood, lymphoid organs, and tumor tissues. Functional analyses further revealed increased IFN-γ and TNF-α production by both CD8⁺ and CD4⁺ T cells, accompanied by a reduction in regulatory T cell frequencies without induction of exhaustion-associated phenotypes. Together, these findings indicate that RMAD1-based vaccination enhances immune activation while maintaining immune balance.

Moreover, the comparison between E7-RMAD1 and cisplatin treatment revealed comparable efficacy in tumor inhibition, with the RMAD1 group exhibiting a more favorable safety profile, as indicated by the stable body weight of the treated animals. Importantly, the efficacy of RMAD1 extended beyond primary tumors, significantly reducing metastatic burden in a stringent TC-1 lung metastasis model. These results highlight the potential of RMAD1 to generate broad, durable, and therapeutically meaningful anti-tumor immunity. This underscores the therapeutic potential of RMAD1 in cancer immunotherapy, offering a safer alternative to traditional chemotherapies while maintaining high therapeutic efficacy 41, 42.

Additionally, the ELISPOT assays indicated that E7-RMAD1 vaccination not only elicited specific T cell responses against the E7 antigen but also triggered antigen spreading, as evidenced by the upregulation of E6 antigen-specific T cells. The establishment of durable immunological memory in the form of long-lasting immune responses against the tumor further supports the potential use of RMAD1 as an immunotherapeutic agent 43, 44. The lack of tumor growth upon re-challenge in our preclinical models highlights the capacity of RMAD1 to induce robust and long-lasting immune memory, a critical feature for effective cancer vaccines.

Despite these promising findings, several limitations should be acknowledged. The current study was conducted exclusively in immunocompetent murine models, which do not fully recapitulate the complexity of the human immune system. To advance toward clinical translation, future studies will evaluate RMAD1-based vaccine platforms in humanized mouse models, enabling assessment of antigen presentation, T cell priming, immune regulation, and safety in a human immune context. Such studies will be critical for validating the translational relevance of RMAD1 with respect to HLA-restricted antigen presentation and human T cell responses.

In conclusion, RMAD1 represents a novel and effective CPP with significant potential in drug delivery and immunotherapy. Its superior ability to enhance cellular uptake, promote antigen delivery, and stimulate robust immune responses makes it an ideal candidate for targeted therapeutic applications. Future studies should focus on further elucidating the mechanisms underlying cellular uptake, optimizing its conjugation with various therapeutic agents, and evaluating its efficacy in other disease models. As the demand for more efficient and safer therapeutic delivery systems grows, RMAD1 offers a promising platform for the development of next-generation vaccines and immunotherapies.

## Methods

### Mice

Female C57BL/6 mice aged 7 weeks were purchased from Samtako (Osan, Korea) and housed in the animal facility at the College of Pharmacy, Sahmyook University. All animal experiments were conducted in accordance with protocols approved by the Sahmyook University Institutional Animal Care and Use Committee (Approval no. SYUIACUC 2024-012).

### Therapeutic mouse model

For the therapeutic cancer vaccine model, 1 x 106 E.G7-OVA cells were subcutaneously injected into the right flank of C57BL/6 mice. Vaccines containing either OVA-SLP or OVA-RMAD1 (3.9 nmol) were administered at the dorsal side opposite to the tumor site on days 3 and 10 post-tumor inoculation. For the TC-1 tumor model, 1 × 105 TC-1 cells were injected subcutaneously, and mice received vaccinations with either E743-62 (GQAEPDRAHYNIVTFCCKCD) or its RMAD1-conjugated form (8.8 nmol) on days 6 and 13. Tumor volume was measured using digital calipers and calculated by following formula (0.52 × length × width2). All animals were randomly allocated before treatment and were euthanized when tumor size exceeded 1500 mm3. To establish a lung metastasis model, C57BL/6 mice were intravenously injected with 4 × 10⁵ TC-1 cells via the tail vein. Mice were vaccinated with E7-RMAD1 subcutaneously on days 6 and 13 after tumor challenge. At day 28, mice were euthanized and lungs were harvested for evaluation of metastatic burden. Lung weight was measured for indirect marker of metastatic lung burden and metastatic nodules were counted.

### Flow cytometry analysis

Tumors, spleens, draining lymph nodes, and peripheral blood were collected for analyzing immune cell populations. Tumor tissues were enzymatically dissociated by a tumor dissociation kit (Miltenyi Biotec) while spleens and lymph nodes were mechanically dissociated to generate single cells. Following red blood cell lysis, all samples were processed for flow cytometry. Cells were stained with antibodies against CD8, CD3, OVA (H-2Kb-SL8) tetramer and CD45 at 4 °C for 30 min. For intracellular cytokine staining, cells were re-stimulated with E749-57 (RAHYNIVTF) peptides to assess antigen specific CD8⁺ T cell responses, or with E743-62 SLP to evaluate antigen specific CD4⁺ T cell responses. BD Fixation/permeabilization kits were used to analyze IFN-γ and TNF-α expression. Regulatory T cells were counted by using antibodies against CD3, CD4, and CD25, followed by intracellular staining with Foxp3 antibodies. T cell exhaustion markers were tested using antibodies against PD-1, LAG-3, and TIM-3 antibodies. All samples were examined using a flow cytometer, and data were analyzed using FlowJo software.

### *In vitro* toxicity assessment

TC-1, DC2.4, and HaCaT cells were used for testing *in vitro* toxicity. Cells were treated with 12.5 μM of RMAD1 for 72 h, and cell viability was assessed using a CCK-8 assay. Cell viability was further tested by using Annexin V/propidium iodide (PI) staining kit to quantify the viable cells.

### *In vivo* toxicity assessment

To test the systemic toxicity, C57BL/6 mice were injected with control, cisplatin, Pam2CSK4, or RMAD1-conjugated vaccines. Body weight was monitored during the treatment period and spleen weights were normalized to body weight to assess splenomegaly. After collecting serum from the mice, cytokines including IL-6 and IL-12 were measured and organ toxicity was evaluated by checking alanine aminotransferase, aspartate aminotransferase, blood urea nitrogen, and lactate dehydrogenase levels.

### *In vitro* cross-presentation assay

DC2.4 cells were seeded at 1 × 105 cells in 96-well plates and incubated with OVA-RMAD1 with or without MPLA for 16 h. After PBS washing, B3Z reporter cells were added at 2 × 105 cells/well. After co-culture, cells were centrifuged, washed, and incubated with a CPRG substrate solution (containing 0.5% NP-40 and CPRG) at 37 °C for 4 h. Absorbance at 570 nm (reference 650 nm) was measured to quantify B3Z activation.

### Elispot assay

IFN-γ production was assessed using pre-coated ELISpot plates (Mabtech, #3321-4AST-10). Plates were blocked with RPMI + 10 % FBS for 1 h, then 5 × 105 tumor-draining lymph node cells were seeded per well. Cells were stimulated with 2 μg/mL of HPV16 E6 (5-25 aa) or E7 (43-62 aa) peptides for 24 h. After extensive washing, plates were incubated sequentially with biotinylated anti-IFN-γ antibody, streptavidin-ALP, and developed with BCIP substrate. Spot counts were analyzed using an AID ELISpot reader and software.

### Biodistribution

Mice were subcutaneously administered 25 nmol of FITC-labeled OVA-RMAD1 along with MPLA. After 24, 48, and 72 h, major organs were harvested, washed with PBS, and analyzed using an *In vivo* Imaging System Lumina II imaging systems and living image software.

### Peptide selection and synthesis

Candidate peptides were selected using the intra-dermal delivery technology (IDDT) platform based on predefined scoring criteria. Peptides exceeding the reference threshold defined by the CPP score of TAT were chosen for synthesis. Peptides including CPPs, RMAD1 derivatives, and SLP-CPPs were synthesized by Fmoc solid-phase peptide synthesis (SPPS) on a chloride resin. Following cleavage and deprotection using TFA-based cocktails, peptides were purified via reverse-phase HPLC with a C18 column and characterized by LC/MS analysis. For antigen-CPP conjugates, peptides were synthesized as single sequences using Fmoc-based SPPS, with a glycine-glycine (GG) linker introduced between the antigen and RMAD1 to provide structural flexibility. The sequences of all synthesized peptides are provided (Table S1).

### Western blot analysis

HaCaT cells (3 x 105) were seeded and treated with CPP-recombinant proteins for 2 h. Cells were lysed with RIPA buffer (Biosesang, Seongnam, Korea) containing protease inhibitors, and lysates were cleared by centrifugation. 20 μg of protein was separated by SDS-PAGE, transferred to PVDF membranes, and blocked with 5 % skim milk in TBS-T, then probed with anti-GFP and anti-tubulin antibodies, followed by HRP-conjugated secondary antibodies. Signals were developed using ECL reagent and captured using X-ray film or a LuminoGraph2 imaging system.

### Penetration assay

DC2.4, HaCaT and TC-1 cells were incubated with FITC-labeled peptides or proteins for 2 h. After washing and trypsinization to remove surface-bound molecules, internalized peptides were assessed via flow cytometry. To distinguish surface-bound signals from internalized fluorescence, selected experiments were performed with additional trypan blue treatment to quench extracellular signals prior to analysis. For quantitative assessment of uptake efficiency, a six-point dose response penetration assay was performed in HaCaT cells, and EC₅₀ values were calculated based on mean fluorescence intensity (MFI). To evaluate the temperature dependence of cellular uptake, penetration assays were additionally performed at 4 °C and 37 °C in DC2.4, HaCaT, and TC-1 cells. Cells were pre-equilibrated at the indicated temperatures prior to incubation with FITC-labeled peptides, and internalization efficiency was compared between conditions. To study endocytic pathways, DC2.4 cells were pretreated with endocytosis inhibitors (CPZ, MBCD, or Amiloride) prior to CPP exposure. Localization in HaCaT cells was visualized using fluorescence microscopy after Hoechst nuclear staining 45.

### ELISA

RAW264.7 cells were seeded at a density of 5 × 104 cells/ml in 24-well plates. The next day, the medium was replaced with serum-free media for 1 h prior to stimulation. Cells were then treated with varying concentrations of RMAD1 for 24 h. Following treatment, culture supernatants were collected by centrifugation at 500 g for 10 min and analyzed for cytokine production using a mouse IL-6 ELISA set (BD, USA).

### Protein purification

Recombinant EGFP-CPP fusion proteins and SOD1-CPP constructs were expressed using pET-28a and pET-29b vector systems, respectively. Plasmids were transformed into E. coli BL21 (DE3) cells, and single colonies were expanded until reaching optical density at 600 nm of 0.5. Protein expression was induced with 0.5 mM IPTG (EMD Millipore) at 4 °C for 16 h. Bacterial pellets were harvested and lysed by sonication in Tris buffer containing 300 mM NaCl. After removing debris by centrifugation at 20,000 g for 30 min, the supernatant was loaded to a column, washed with 50 mM Tris, pH 7.5, containing 300 mM NaCl, 5 % glycerol, and 15 mM imidazole and the proteins were eluted with buffer (50 mM Tris pH 7.5, 300 mM NaCl, Glycerol 5%, 300 mM imidazole). Endotoxins were removed using mustang column (Sigma-Aldrich) and protein samples with endotoxin levels below 1 EU/mg, as assessed by the LAL assay (Thermo Fisher Scientific) were used for subsequent experiments.

### Circular dichroism spectroscopy

Circular dichroism spectra were recorded using an Applied Photophysics spectropolarimeter at 298 K. Measurements were performed over a wavelength range of 190-260 nm using a quartz cuvette with a 0.5-mm path length. Peptides were dissolved at the 0.2 mg/mL in either distilled water or 50 % (v/v) 2,2,2-trifluoroethanol (TFE) to assess secondary structure under aqueous and membrane-mimicking conditions. The acquired spectra were processed and visualized using GraphPad Prism.

### Statistics

Results are presented as mean ± standard deviations and statistical significance was obtained with student's t-tests for pairwise comparisons between groups. P < 0.05 was considered statistically significant for differences between the two groups. Statistical analysis of survival and tumor-free status was performed using Kaplan-Meier analysis with the log-rank (Mantel-Cox) test.

## Supplementary Material

Supplementary figures and table.

## Data Availability

All data relevant to the study are included in the article.
