# Supplementary material for: RMAD1, a Novel Cell-Penetrating Peptide Derived from ADARB2: Preclinical Insights into Antigen Uptake and T Cell Activation
Source: Int J Biol Sci. 2026 Jun 4;22(11):6084–99. doi: 10.7150/ijbs.132948 (PMC13282796; doi:10.7150/ijbs.132948)
Supplement: Supplementary file 1 — Supplementary figures and table. [file ijbsv22p6084s1.pdf]

RMAD1, a Novel Cell-Penetrating Peptide Derived from ADARB2: Preclinical Insights into Antigen Uptake and T Cell Activation

Chaemin Lim[1,#], Chanho Park[2,#], Ee Chan Song[2], Yungyeong Shin[2], Wan Ki Kim[2], Yu Jin Park[2], Hyejoo Youn[1], Cheolmin Ham[3], Sang Bum Kim[4], Seongmin Cho[2,*]

[1] *College of Pharmacy, CHA University, 335 Pangyo-ro, Bundang-gu, Seongnam-si, 13488 Gyeonggi-do, Republic of Korea*

[2] *Remedi Co., Ltd. Research center, Songdo 21990, Korea*

[3] *Institute for Rare Isotope Science, Institute for Basic Science, Daejeon 34000, Republic of Korea*

[4]*College of Pharmacy, Sahmyook University, Seoul 01795, Korea.*

[#] Those authors contributed equally to the manuscript.

*Correspondence: Seongmin Cho, Ph.D. Remedi Research Center, Songdo 21990, Korea (Tel: 82-32-819-3399, Fax: 82-32-819-3398, E-mail: scho@re-medi.co.kr)

Supplementary Figures

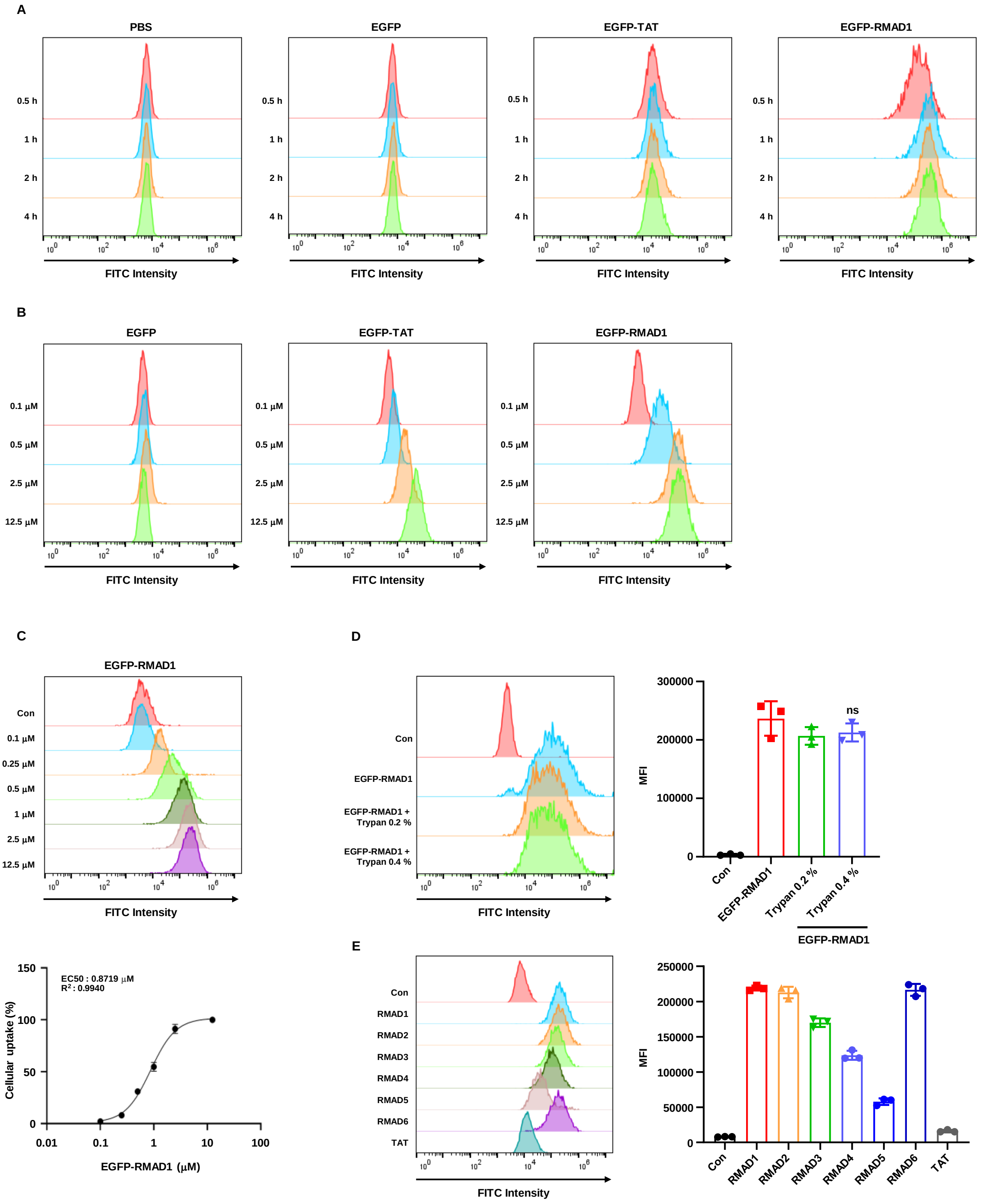

Figure S1

Superior cellular penetration of RMAD1 compared with other CPPs in HaCaT cells. (A) Representative flow cytometry histograms showing the time-dependent intracellular uptake of EGFP-RMAD1 in HaCaT cells, quantified as mean fluorescence intensity (MFI). (B) Representative histograms illustrating the dose-dependent cellular penetration of EGFP-RMAD1 in HaCaT cells. (C) Dose–response curve showing the EC_{50} value for EGFP-RMAD1 cellular penetration in HaCaT cells, together with representative histograms at the indicated concentrations (n=3 per group). (D) Trypan blue quenching assay showing intracellular localization of EGFP-RMAD1 (2.5 μM), confirming that the detected fluorescence originates from internalized peptides rather than membrane-associated signals. (E) Cellular uptake analysis of RMAD1-derived and sequence-modified variants (2.5 μM), demonstrating reduced internalization efficiency compared to the original RMAD1 peptide. Data are presented as mean ± SD.

A

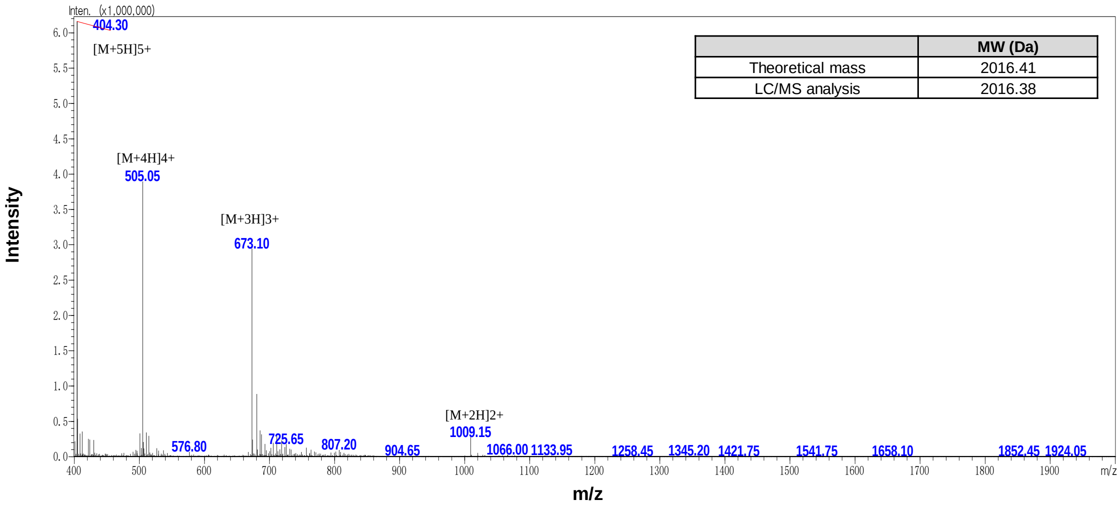

B

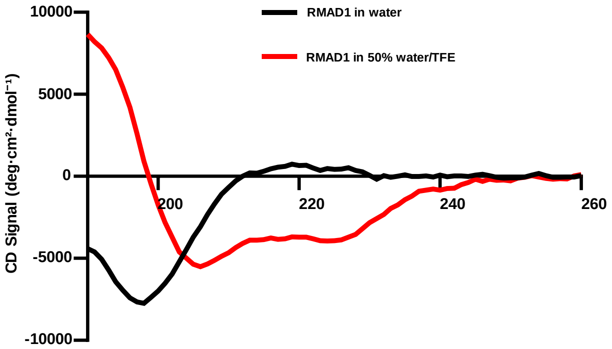

Figure S2

Structural characterization of the RMAD1 peptide. (A) Mass spectrometry analysis confirming the molecular weight of RMAD1, consistent with the calculated mass. (B) Circular dichroism spectra of RMAD1 (0.2 mg/mL) in aqueous solution and in the presence of 50 % TFE, showing increased α-helical content under membrane-mimicking conditions.

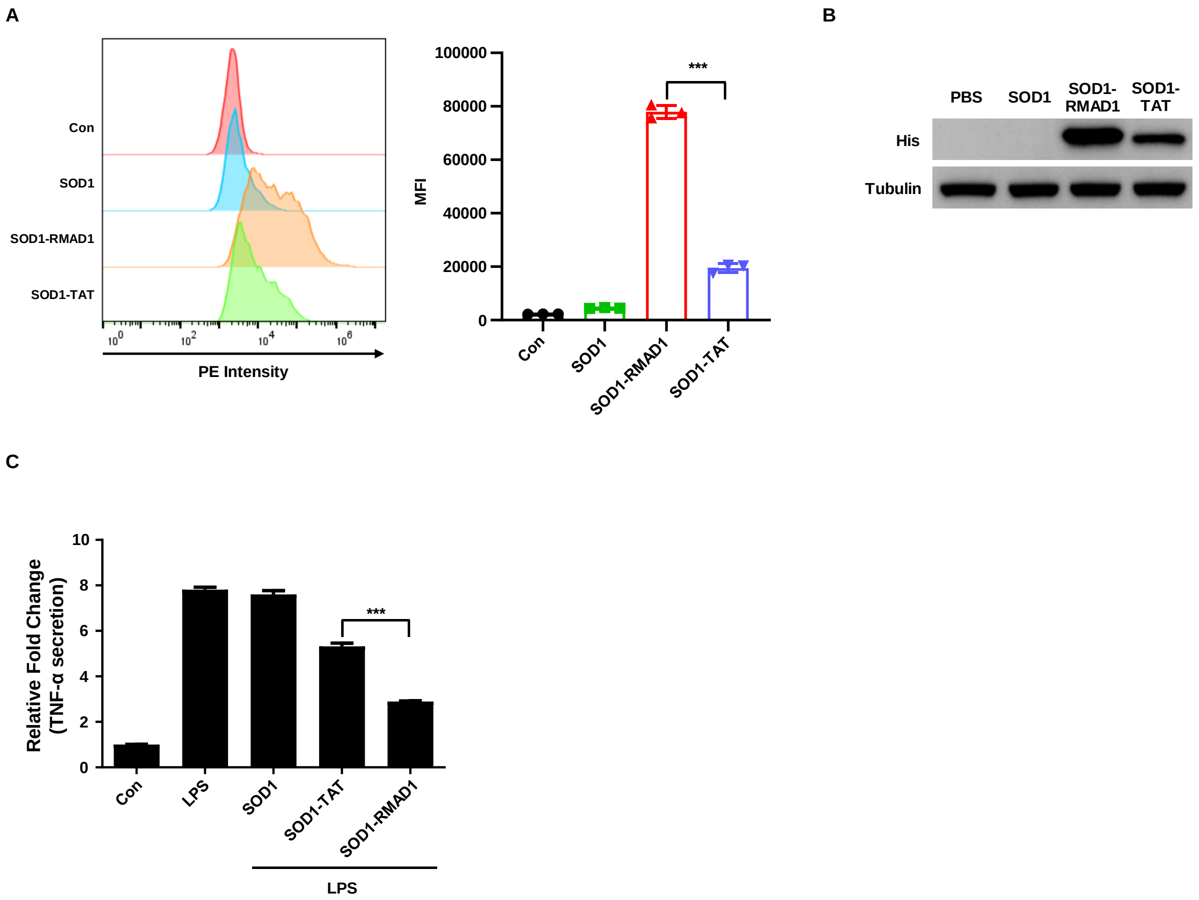

Figure S3.

RMAD1-mediated intracellular delivery and functional validation of SOD1. (A) Flow cytometry analysis of intracellular uptake of TRITC-labeled SOD1 (2.5 μM) in DC2.4 cells, demonstrating enhanced delivery in the presence of RMAD1. (B) Western blot analysis confirming increased intracellular accumulation of SOD1–RMAD1 (2.5 μM) compared with control groups. (C) Functional activity of delivered SOD1 assessed in an LPS-induced inflammatory model. DC2.4 cells were stimulated with LPS (500 ng/mL) and treated with SOD1–RMAD1 (2.5 μM), followed by measurement of TNF-α secretion using ELISA, showing a significant reduction compared to controls. Data are presented as mean ± SD, and statistical significance was determined using Student's *t*-test (*** $p < 0.001$).

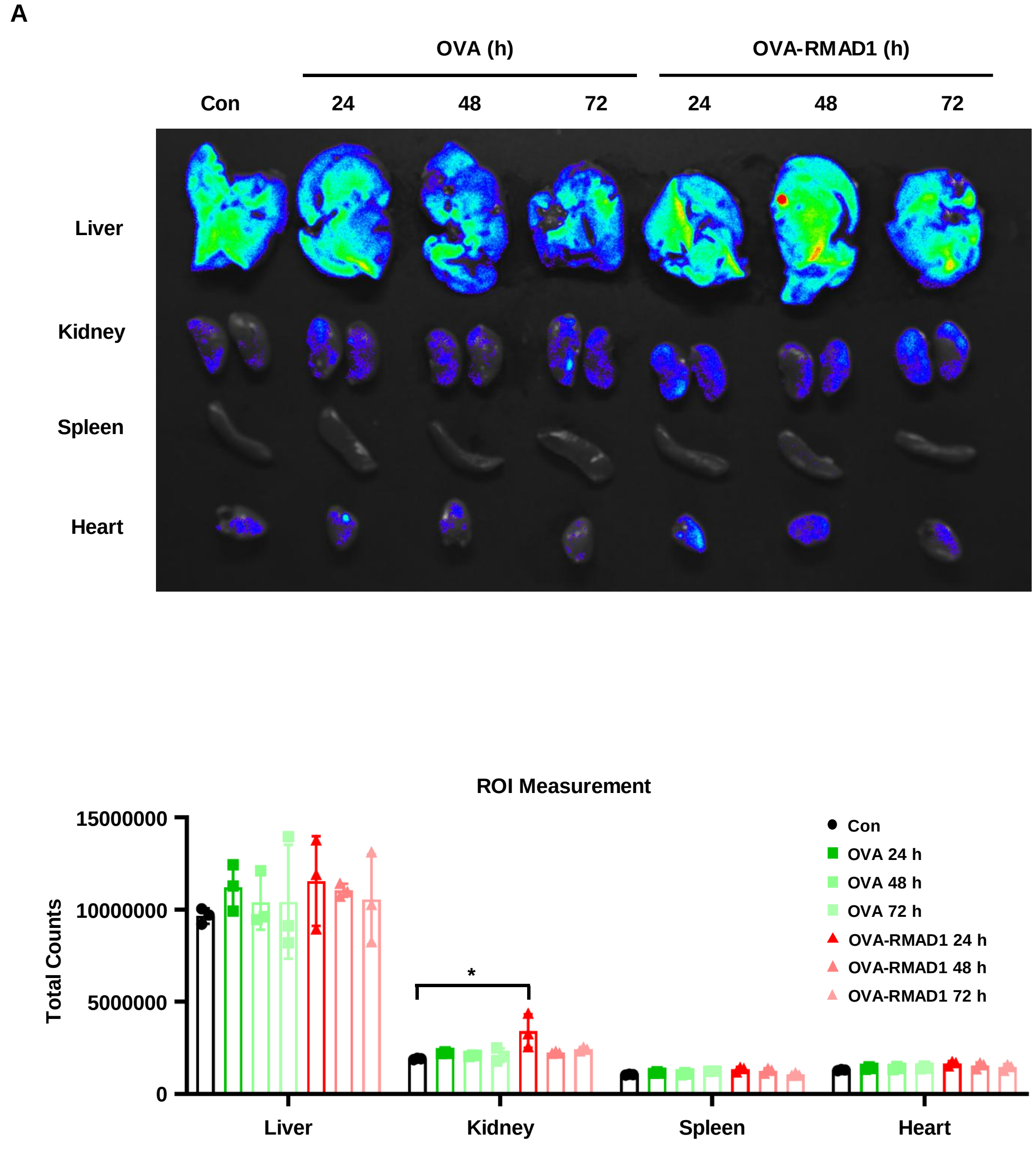

Figure S4

Organ biodistribution and clearance profile of OVA–RMAD1**.** (A) C57BL/6 mice were subcutaneously administered 25 nmol of FITC-conjugated OVA–RMAD1. At 24, 48, and 72 h post-administration, major organs, including the liver, kidney, spleen, and heart, were harvested and analyzed by IVIS imaging (n=3 per group). Fluorescence signals in each organ were quantified as total fluorescence counts to evaluate the time-dependent biodistribution and clearance of OVA–RMAD1. Data are presented as mean ± SD, and statistical significance was determined using Student's *t*-test (* $p < 0.05$).

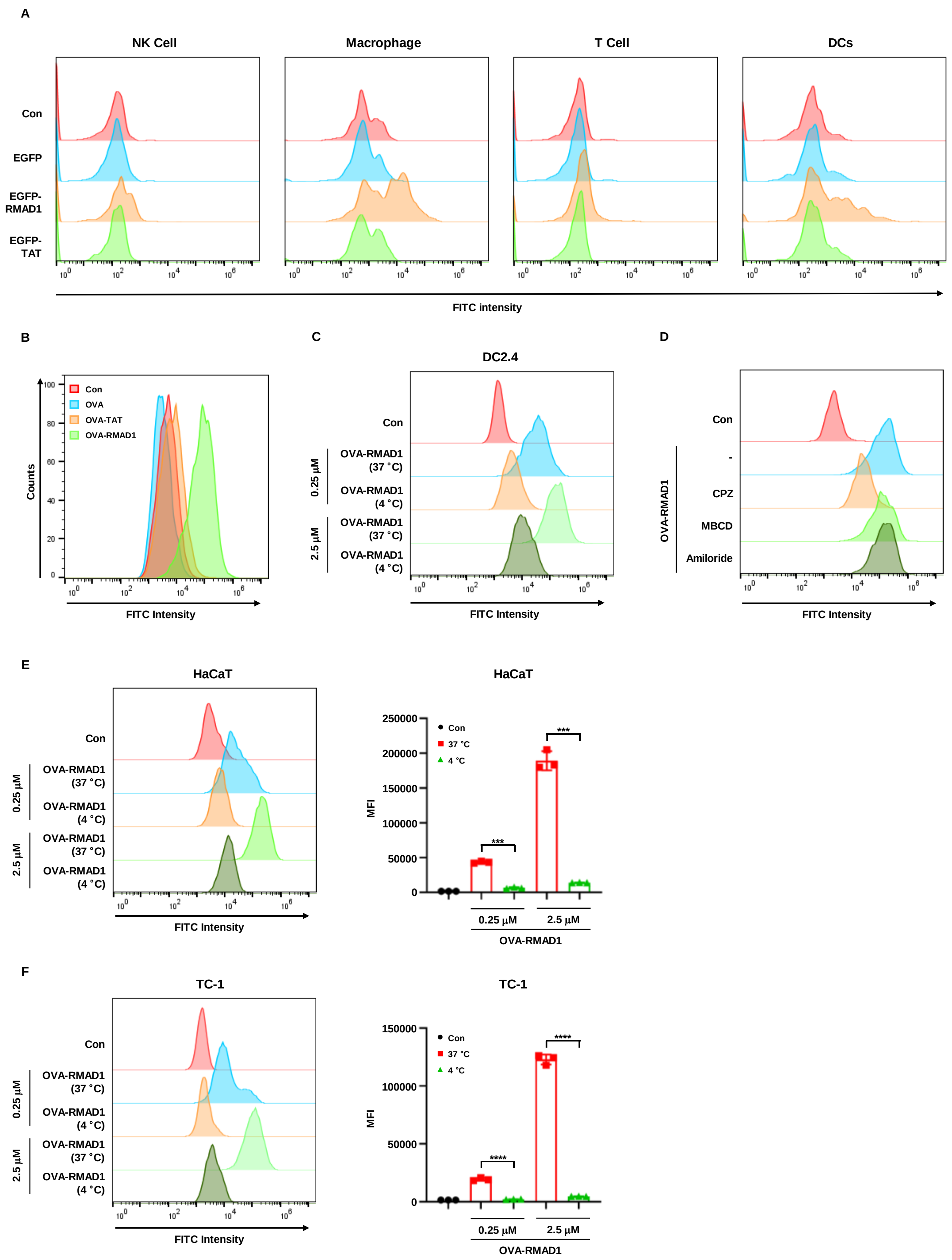

A
NK Cell
Macrophage
T Cell
DCs
Con
EGFP
EGFP-RMAD1
EGFP-TAT
FITC intensity
B
Con
OVA
OVA-TAT
OVA-RMAD1
Counts
FITC Intensity
C
DC2.4
Con
0.25 μM
OVA-RMAD1 (37 °C)
OVA-RMAD1 (4 °C)
2.5 μM
OVA-RMAD1 (37 °C)
OVA-RMAD1 (4 °C)
FITC Intensity
D
Con
OVA-RMAD1
-
CPZ
MBCD
Amiloride
FITC Intensity
E
HaCaT
Con
0.25 μM
OVA-RMAD1 (37 °C)
OVA-RMAD1 (4 °C)
2.5 μM
OVA-RMAD1 (37 °C)
OVA-RMAD1 (4 °C)
FITC Intensity
HaCaT
MFI
Con
37 °C
4 °C
***
***
0.25 μM
2.5 μM
OVA-RMAD1
F
TC-1
Con
0.25 μM
OVA-RMAD1 (37 °C)
OVA-RMAD1 (4 °C)
2.5 μM
OVA-RMAD1 (37 °C)
OVA-RMAD1 (4 °C)
FITC Intensity
TC-1
MFI
Con
37 °C
4 °C
****
****
0.25 μM
2.5 μM
OVA-RMAD1

Figure S5

RMAD1 uptake is mediated by clathrin-dependent endocytosis. (A) Representative flow cytometry histograms corresponding to Fig. 2B, showing RMAD1 uptake in various immune cell populations as assessed by an ex vivo cell-binding assay. (B) Representative histograms corresponding to Fig. 2C, demonstrating the cellular penetration of OVA–RMAD1 in DC2.4 cells. (C) Representative histograms corresponding to Fig. 2D, showing RMAD1 uptake following treatment at 0.25 or 2.5 μM under 4 ℃ or 37 ℃ conditions, indicating energy-dependent internalization. (D) Representative histograms comparing OVA–RMAD1 uptake in DC2.4 cells following treatment with various endocytosis inhibitors, demonstrating involvement of clathrin-mediated endocytosis**.** (E, F) Quantitative analysis and representative histograms of RMAD1 uptake in HaCaT (E) and TC-1 (F) cells across different concentrations under 4 ℃ and 37 ℃ conditions (n=3 per group). Data are presented as mean ± SD, and statistical significance was determined using Student's *t*-test (*** $p < 0.001$).

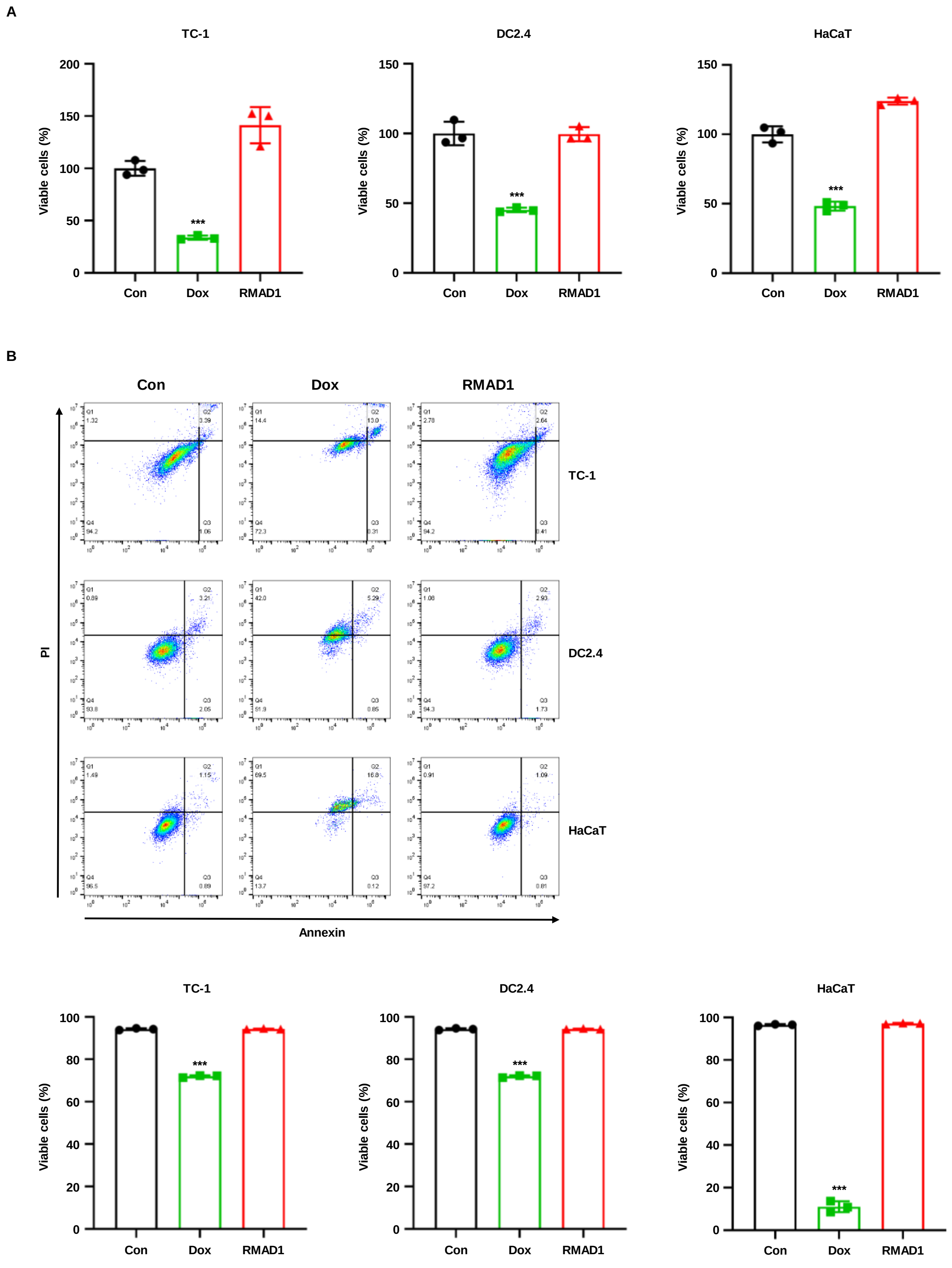
A
TC-1
DC2.4
HaCaT
Viable cells (%)
Con
Dox
RMAD1
***
B
Con
Dox
RMAD1
TC-1
DC2.4
HaCaT
PI
Annexin
TC-1
DC2.4
HaCaT
Viable cells (%)
Con
Dox
RMAD1
***

Figure S6

RMAD1 exhibits excellent safety profiles across multiple cell lines. (A) In vitro cytotoxicity of RMAD1 was evaluated at 72 h following treatment with 12.5 μM RMAD1 using a CCK-8 assay in TC-1, DC2.4, and HaCaT cells. Doxorubicin was used as a positive control for cytotoxicity (n=3 per group). (B) The proportion of viable cells following treatment with 12.5 μM RMAD1 was quantified using Annexin V/propidium iodide (PI) staining in TC-1, DC2.4, and HaCaT cells (n=3 per group). Representative dot plots corresponding to the Annexin V/PI analysis are shown. Data are presented as mean ± SD, and statistical significance was determined using Student's *t*-test (*** $p < 0.001$).

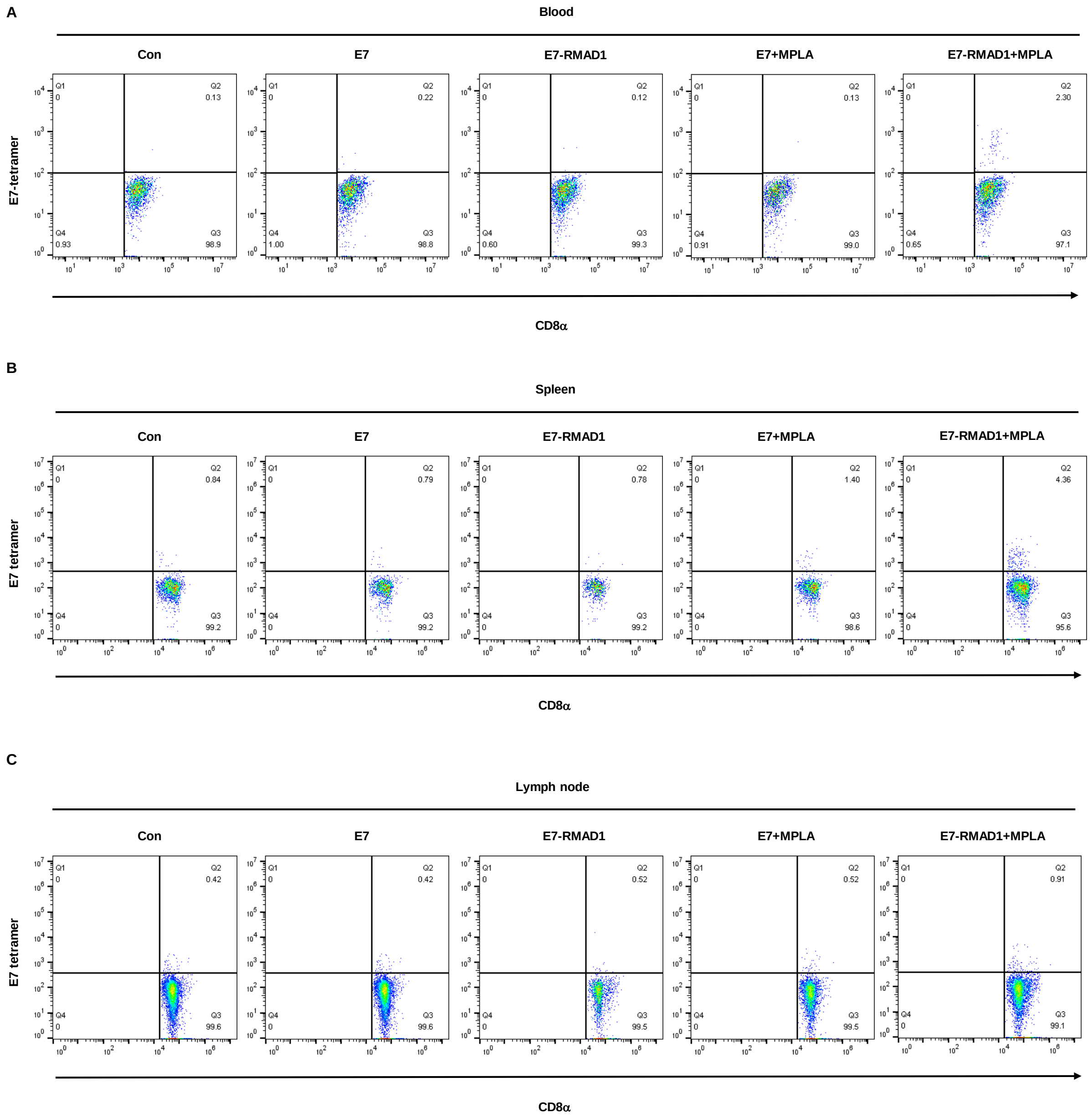

Figure S7

Conjugation of E7 SLP to RMAD1 enhances antigen-specific T cell responses**.** (A) Representative flow cytometry dot plots corresponding to Fig. 4C, showing the frequency of tetramer-positive T cells in peripheral blood. (B, C) Representative dot plots corresponding to Fig. 4D and 4E show tetramer-positive T cells in the spleen (B) and lymph nodes (C)**,** respectively.

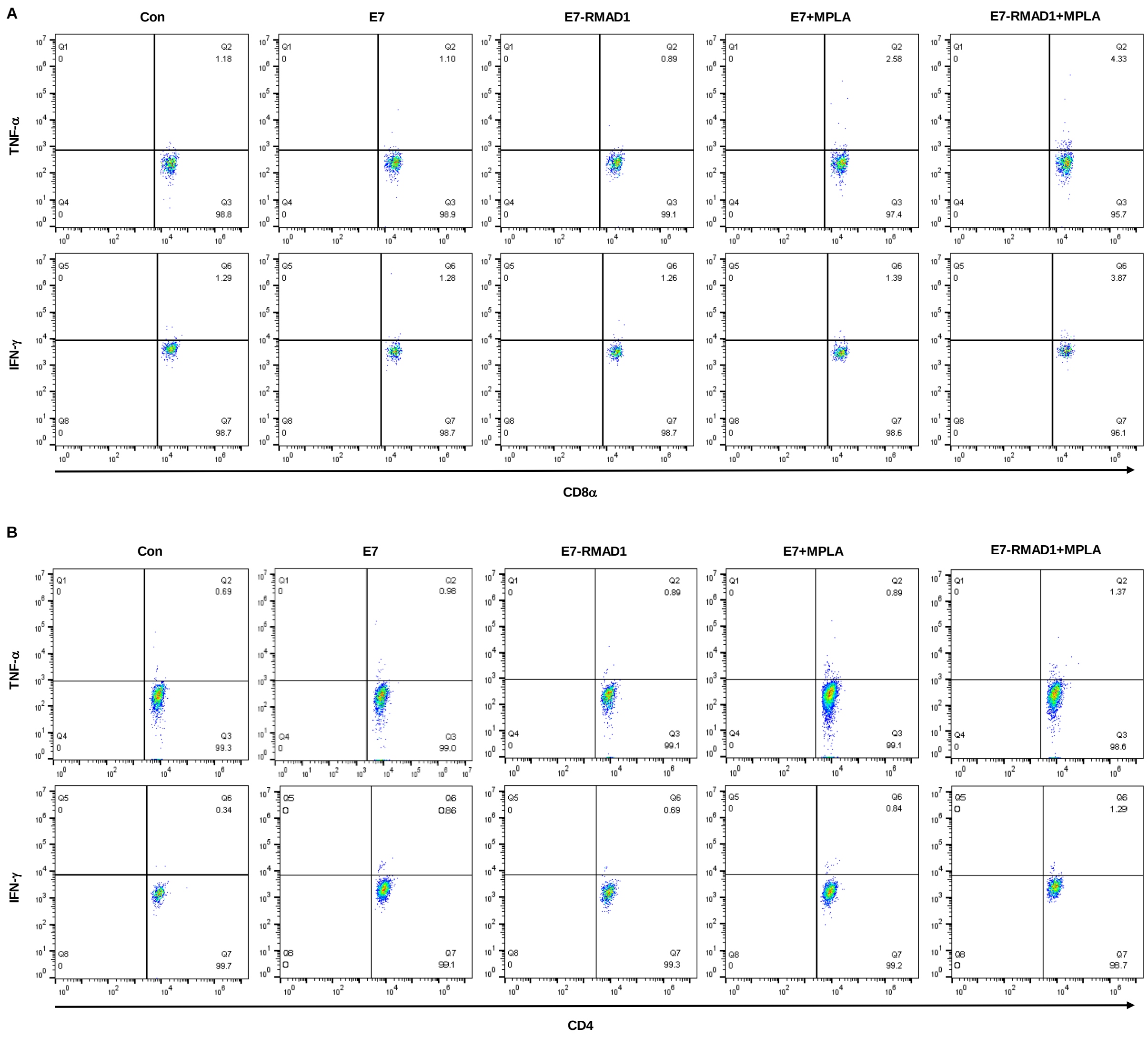

Figure S8

Conjugation of E7 SLP to RMAD1 enhances functional T cell responses. (A, B) Representative dot plots corresponding to Fig. 4F and 4G, showing the frequencies of TNF-α– and IFN-γ–producing CD8^{+} (A) and CD4^{+} T (B) cells.

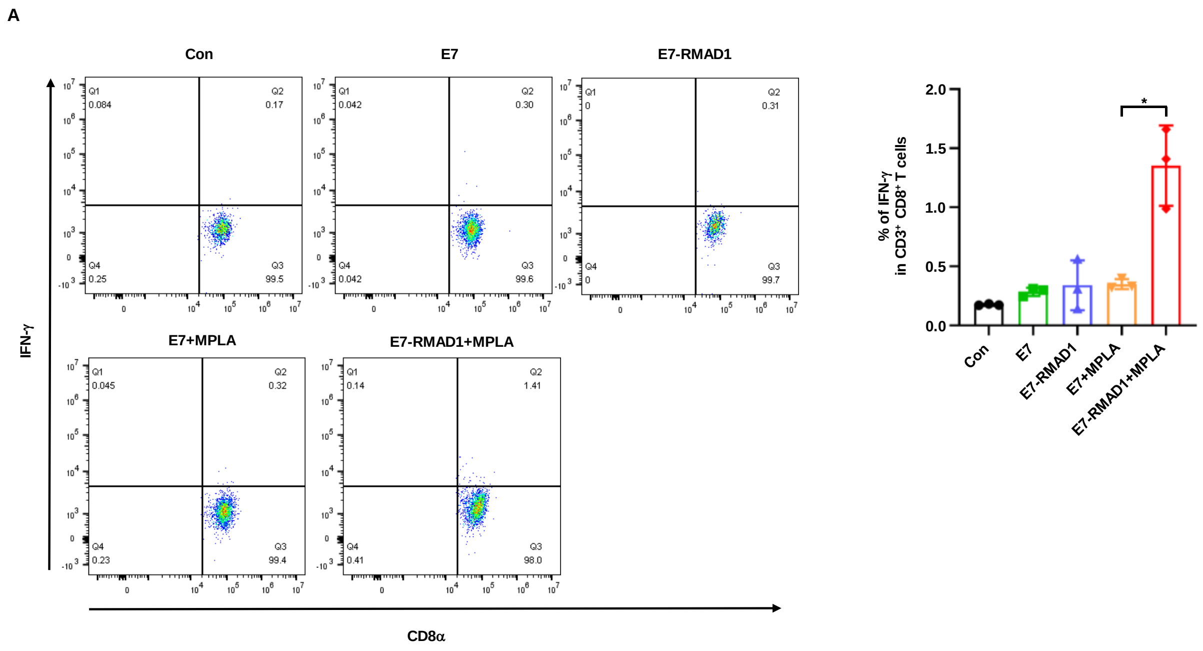

Figure S9

Conjugation of E7 SLP to RMAD1 enhances functional T cell responses in tumors. (A) Tumors were harvested, and the proportion of IFN-γ^+ $CD8^+$ T cells was quantified by intracellular staining and representative dot plots are shown (n=3 per group). Data are presented as mean ± SD, and statistical significance was determined using Student's *t*-test (* $p < 0.05$).

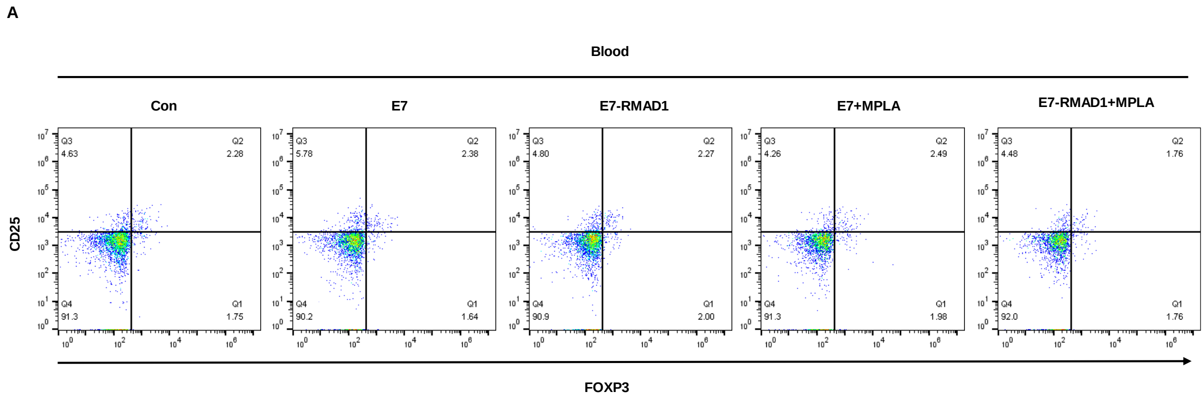

Figure S10

Conjugation of E7 SLP to RMAD1 reduces population of Tregs in blood. (A) Representative dot plots corresponding to Figure 4H, showing the frequency of $Foxp3^+CD25^+$ regulatory T cells in peripheral blood.

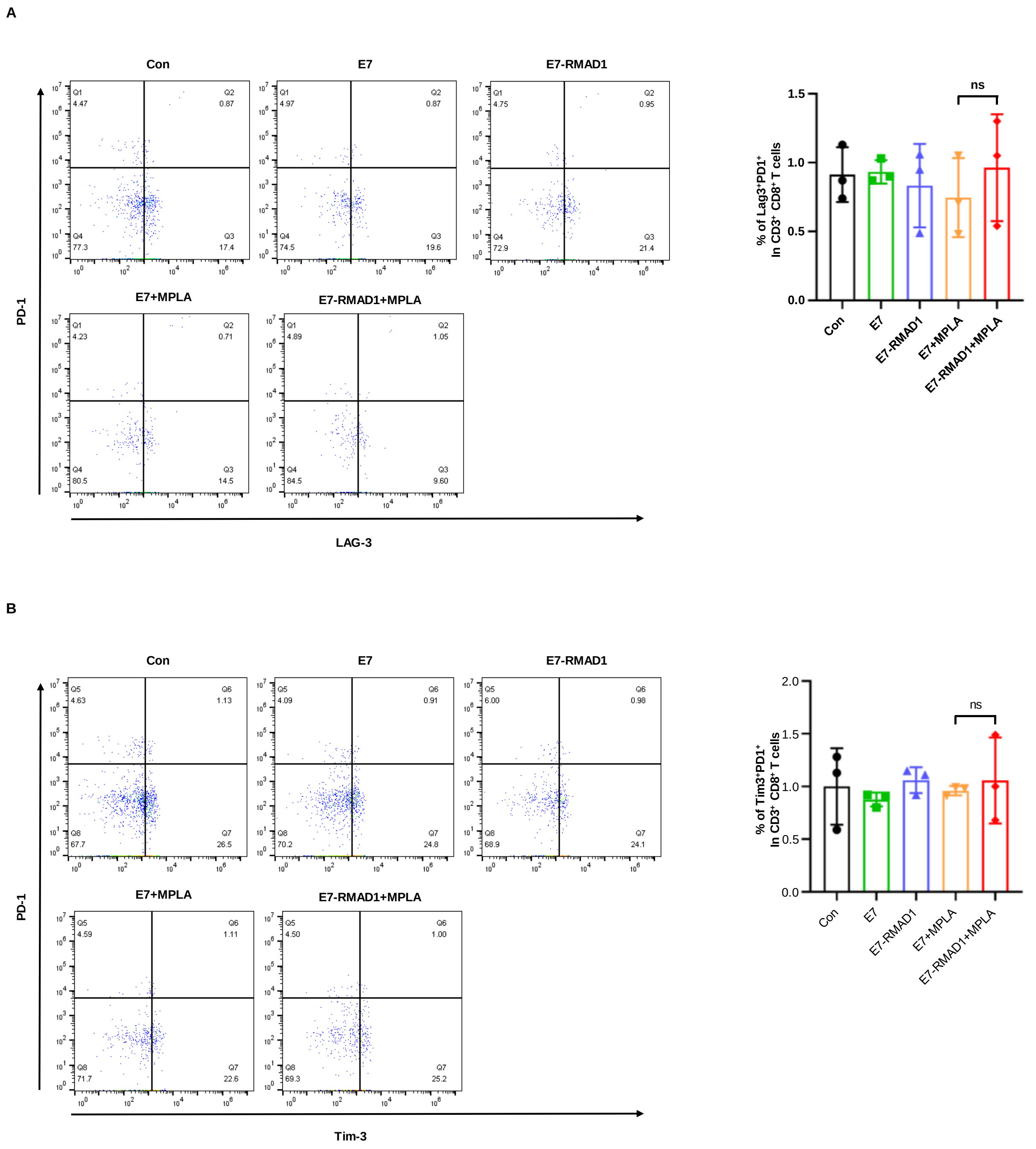

Figure S11

Assessment of CD8^{+} T-cell exhaustion. (A) The extent of CD8^{+} T-cell exhaustion was evaluated based on the frequency of PD-1^{+}LAG-3^{+} cells (n=3 per group). Quantitative graphs and representative flow cytometry dot plots are shown. (B) CD8^{+} T-cell exhaustion was further assessed by the frequency of PD-1^{+}TIM-3^{+} cells (n=3 per group). Quantitative graphs and representative flow cytometry dot plots are shown. Data are presented as mean ± SD.

Peptide name	Sequence
OVA	DEVSGLEQLESIINFEKLAAAAAK
OVA-RMAD1	DEVSGLEQLESIINFEKLAAAAAKggCKSKRRRRRRSKRKD
OVA-TAT	DEVSGLEQLESIINFEKLAAAAAKggYGRKKRRQRRR
E7	GQAEPDRAHYNIVTFCCKCD
E7-RMAD1	GQAEPDRAHYNIVTFCCKCDggCKSKRRRRRRSKRKD
RMAD1	CKSKRRRRRRSKRKD
RMAD2	CRSKRRRRRRSRRRD
RMAD3	CKSKKKKKKKSKRKD
RMAD4	CKSKRRRRSKR
RMAD5	SKRRRRSKRD
RMAD6	YKSKRRRRRRSKRKD
TAT	YGRKKRRQRRR

Table S1.

Peptide sequences used in this study. Table summarizing the names and amino acid sequences of peptides utilized in this study.
